# Molecular design principles for bipolar spindle organization by two opposing motors

**DOI:** 10.1073/pnas.2422190122

**Published:** 2025-03-21

**Authors:** Wei-Xiang Chew, François Nédélec, Thomas Surrey

**Affiliations:** ^a^Centre for Genomic Regulation, The Barcelona Institute of Science and Technology, Barcelona 08003, Spain; ^b^Sainsbury Laboratory, University of Cambridge, Cambridge CB2 1LR, United Kingdom; ^c^Universitat Pompeu Fabra, Barcelona 08010, Spain; ^d^Catalan Institution for Research and Advanced Studies (ICREA), Barcelona 08010, Spain

**Keywords:** microtubule network, motor proteins, bipolar spindle, active matter, computer simulation

## Abstract

During cell division in animal cells, a bipolar spindle assembles to segregate the chromosomes. Various motor proteins with different properties are essential for spindle self-organization. The minimal set of components required to organize dynamic microtubules into a bipolar network remains however unknown. Here, we use computer simulations to explore whether two types of microtubule-crosslinking motors with opposite directionality can organize dynamic microtubules into bipolar spindles in three-dimensional space around a local microtubule nucleation source. We find that two motors are indeed sufficient, provided their properties resemble the main human spindle motors kinesin-5 and dynein, revealing the core mechanism of spindle self-organization. It is based on the synergistic interplay of a slow plus-directed symmetric motor and a fast minus-directed asymmetric motor. A hypothetical symmetric minus-directed motor can also support spindle formation together with kinesin-5, but only in a limited and unphysiological parameter range. In agreement with its accessory role in human cells, a minus motor with human kinesin-14 properties does not assemble stable bipolar spindles together with kinesin-5. These results reveal fundamental principles for the self-organization of dynamic bipolar microtubule architectures and highlight how distinct molecular designs of mitotic motors are optimized for their task.

## Significance

When cells divide, the mitotic spindle segregates the genetic material. Correct bipolar spindle assembly depends on mitotic motors with distinct properties. How motors collectively organize microtubules into a bipolar network is not understood. Here, we use computer simulations to explore the core mechanism of motor-driven spindle self-organization. We find that two motors, a plus and a minus motor with appropriate properties, are sufficient to organize dynamic microtubules into a bipolar network. Our work identifies a simple recipe for motor-driven spindle organization and suggests an explanation as to why the main spindle motors in animals, kinesin-5 and dynein, have evolved the properties they have.

During eukaryotic cell division a bipolar spindle self-organizes around chromosomes to segregate the genetic material to the two daughter cells. The spindle is a highly interconnected, active network of microtubules, motors, crosslinkers, and other associated proteins (1, 2). In animal cells, microtubules nucleate in the vicinity of chromosomes and from centrosomes (3, 4). Microtubules are then organized by various microtubule crosslinking motors and passive crosslinkers so that microtubule minus ends become focused at the two spindle poles while the dynamically growing and shrinking plus ends form central antiparallel microtubule overlaps (2, 3). Microtubule bundles interacting with kinetochores on chromosomes, called kinetochore fibers, are integrated in the spindle network (5). In many cells, spindle microtubules slowly flux outward toward the poles, having a lifetime of around a minute while spindles are much longer-lived, emphasizing the highly dynamic nature of this microtubule network (6).

Self-organization of the bipolar microtubule network architecture itself, however, does not strictly depend on the presence of chromosomes, centrosomes, and/or kinetochore fibers, as demonstrated by spindle reconstitutions in *Xenopus* egg extract and laser ablation experiments in cells (4, 7). However, in all animal spindles a slow plus-directed motor, usually a kinesin-5, and the fast minus-directed motor dynein are required for correct spindle assembly, supported by minus-directed kinesin-14 and other motors (8–13). Moreover, the dynein binding microtubule-associated protein NuMA is also required for spindle pole focusing (14–16).

The minimal set of components able to organize dynamic microtubules into a network with a bipolar steady-state architecture is still unknown. Biochemical in vitro reconstitution experiments with purified proteins and computer simulations of minimal reconstituted systems have provided insight into the fundamental microtubule organizing properties of motor proteins. Mixtures of microtubules and a single type of crosslinking motor were shown to organize microtubules either into asters with a focused pole, into nematic networks of extensile bundles or more complex networks, depending on microtubule and motor concentrations, microtubule growth and motor speeds, and crowding conditions (17–20).

Few studies exist with mixtures of two types of motors with opposite directionality. Artificial plus-directed kinesin-1 motor oligomers and minus-directed kinesin-14 motor oligomers generated a network of polarity-sorted microtubules with alternating plus and minus poles (21). Both motors were able to focus microtubule ends into poles, because they were both fast enough to reach their respective microtubule ends where they remained bound for some time. In contrast, mixtures of the natural spindle motor kinesin-5 and natural kinesin-14 generated either nematic networks of extensile bundles or isolated asters with minus poles, resembling the microtubule organization either in the spindle center or at the poles, respectively (22). The emergence of these two types of networks was explained based on the properties of the two opposing motors: Kinesin-5 is normally slower than microtubule growth, unlike kinesin-1 oligomers, and tends to organize microtubules into nematic networks unless microtubules grow very slowly (20). In contrast, kinesin-14 is an asymmetric crosslinker with one motor and one diffusive microtubule binding unit (12, 23), so that it either helps kinesin-5 to bundle microtubules or forms minus poles, depending on which motor dominates (22). So far, no spindle-like network with coexisting antiparallel plus overlaps and focused minus poles has been observed to emerge from mixtures of microtubules and two opposite-directionality motors in biochemical reconstitution experiments.

Spindles and their shape have been described theoretically using mean-field models inspired by liquid crystal physics (24, 25). Alternatively, agent-based computer simulations considering explicitly individual microtubules and motors have successfully produced spindle-like network architectures resembling those in mitotic or meiotic cells (26–31). An important element of models of animal spindles has been a local microtubule nucleation source combined with fast microtubule turnover, mimicking local microtubule nucleation around chromosomes in cells (32). A simple one-dimensional “slide and cluster” model with a kinesin-5-like plus motor and a dynein-like minus motor that was assumed to transport microtubule minus ends only along parallel, but not antiparallel microtubules, could generate steady-state microtubule organizations with two minus poles flanking a region of mixed microtubule polarity (27). How this model would perform in two or three dimensions has however not been tested.

Two-dimensional computer simulations based on more complex models including activities such as autocatalytic, branched microtubule nucleation, repulsive steric interactions between microtubules as well as attractive interactions intended to mimic the effect of a variety of microtubule crosslinkers in addition to explicitly modeling plus and minus motors generated animal spindle-like steady-state architectures (28, 29). In one case, fairly well-focused minus poles, resembling *Xenopus* egg extract spindles, were obtained with a dynein-like motor that transported a NuMA-like oligomerizing microtubule crosslinker and a kinesin-13-like microtubule minus-end depolymerase (29). In the other case, spindles with unfocused poles, resembling mouse oocyte spindles, were obtained with a kinesin-14 instead of a dynein minus motor, together with several other additional spindle activities/components, for example microtubule organizing centers and a microtubule minus end depolymerase (28). The complexity of these previous models and their differences leave it currently unclear, what is the minimal set of components sufficient to organize dynamic microtubules into a bipolar steady-state network.

Using computer simulations, we investigated here whether bipolar spindles can be organized by only two motors with opposite directionality around a local microtubule nucleation source. We find that kinesin-5 and a hypothetical minus-directed symmetric crosslinker can organize microtubules into a stable bipolar spindle, surprisingly via a monopolar intermediate, however only in a narrow parameter range with unphysiologically slow microtubule growth. Kinesin-5 and an asymmetric dynein/NuMA-like microtubule crosslinking motor organize bipolar spindles more robustly, also at faster microtubule growth and via a more natural assembly pathway. Self-organized spindle networks display microtubule flux, like natural spindles. Kinesin-5 and kinesin-14, however, cannot organize stable bipolar spindles. These results emphasize the critical importance of the complementary molecular designs of spindle motors, suggesting an explanation why kinesin-5 and dynein with their distinct properties are the major spindle motors in most animals.

## Model

We simulated active networks of dynamic microtubules and motors using Cytosim, a stochastic cytoskeleton simulator based on Langevin dynamics (22, 33). We focused on identifying a minimal system where motors organize microtubules into bipolar spindles, neglecting many aspects of spindle complexity. Key elements of this system are i) a local source of microtubule nucleation, intended to mimic local microtubule nucleation around chromosomes (32), ii) microtubules with static minus and dynamic plus ends, having a limited lifetime entirely determined by their plus end properties, iii) a plus-directed motor, with properties similar to those measured for human kinesin-5, and iv) a minus-directed motor whose properties were varied to investigate which type of minus motor cooperates best with kinesin-5 to organize the microtubule network into a bipolar spindle architecture. All components are confined within a three-dimensional space allowing microtubules to slide over each other in the presence of repulsive short-range steric interactions between microtubules, resulting in more realistic network behavior than using a two-dimensional model (34). Forces generated by the crosslinking motors move microtubules relative to each other, generating distinct network states. This model has previously reproduced the formation of experimentally observed microtubule networks faithfully (20, 22). For a complete list of simulation parameter values, see *SI Appendix*, Table S1.

## Results

### Kinesin-5 and Kinesin-14 HSET on Their Own Organize Microtubules Around a Local Nucleation Source into Distinct Types of Networks.

**Fig. 1.**
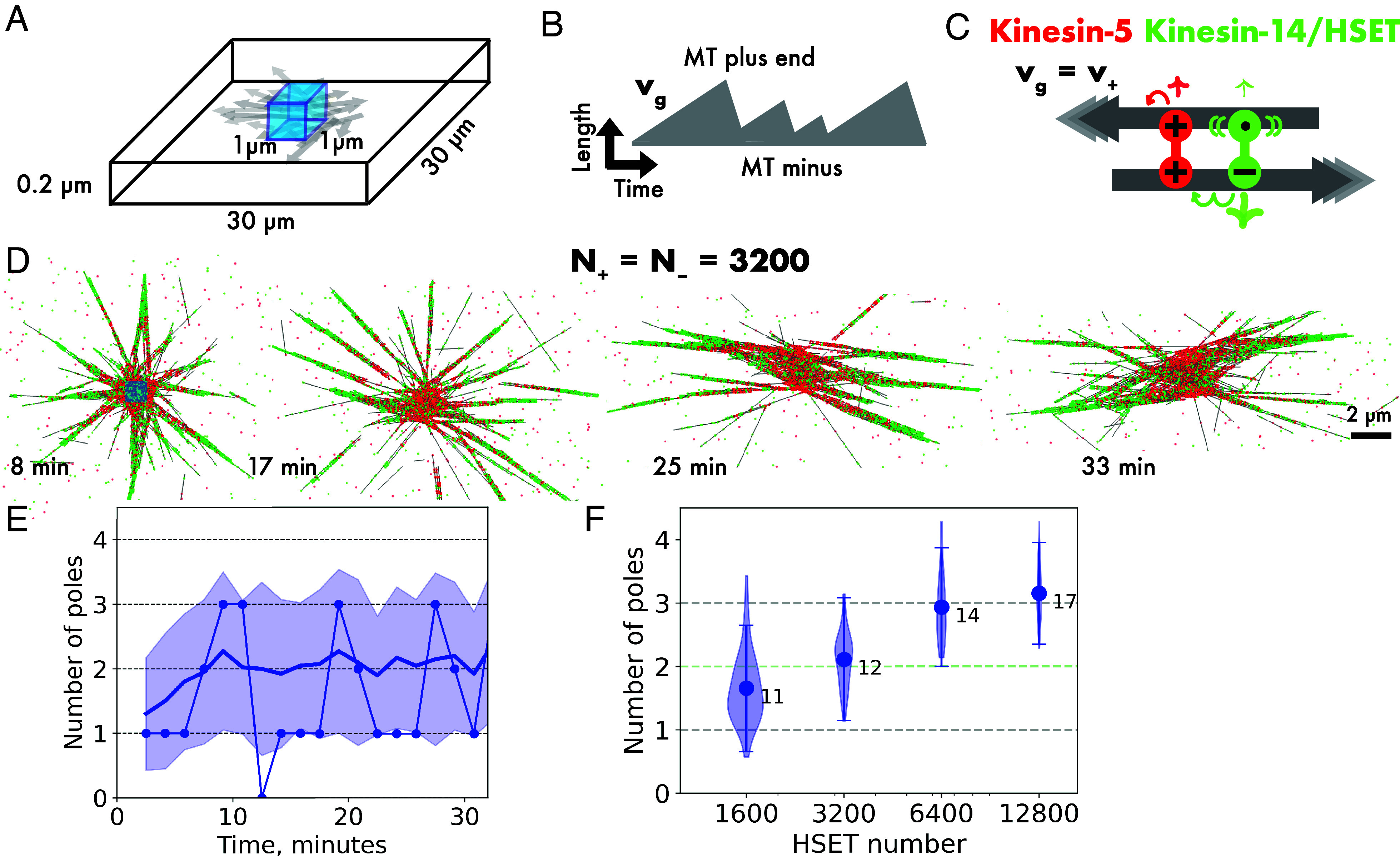
Kinesin-5 and HSET generate microtubule networks with fluctuating numbers of weakly focused poles. (*A*) Schematic of the three-dimensional simulation space (not to scale) with reflective boundaries. At steady state, there are ~358 microtubules. (*B*) Example time course of the length of a microtubule with a static minus and a dynamic plus end. Microtubule growth speed v_g_ = 30 nm/s, average microtubule length L_MT_ = 2.5 µm, lifetime τ_MT_ ~ 1.5 min. (*C*) Schematic of plus-directed motor kinesin-5 (red) and minus-directed motor HSET (green). The plus motor consists of two motor units (symbol +, v_+_ = 30 nm/s, unbinding rate k_off+_ = 0.1/s). The minus motor consists of a minus motor unit (symbol –, v_–_ = 80 nm/s, unbinding rate k_off–_ = 5/s), and a diffusive microtubule binding unit (symbol •, diffusion coefficient = 0.1 µm^2^/s, unbinding rate k_offd_ = 0.01/s). (*D*) Images of a simulated microtubule network organized by N_+_ = 3,200 kinesin-5 and N_–_ = 3,200 HSET motors at the indicated times. The cyan square in the first image indicates the nucleation volume. Only part of the simulation space is shown. (*E*) Time series of the number of detected poles (dots connected by a line) in the network shown in (*D*). The solid line represents the average number of poles, with the shaded region indicating the first SD from the mean. (*F*) Violin plot of the number of poles (shaded area), mean value (circle), average SD of pole number fluctuations over time (error bars), and the average number of microtubules per pole (numbers next to circles) in simulated networks at steady-state organized by N_+_ = 3,200 kinesin-5 and N_–_ = 1,600 to 12,800 HSET motors. Forty simulations were performed for each condition. For a summary of parameters, see *SI Appendix*, Table S2.

First, we established how spindle motors that were studied previously in systems with global microtubule nucleation (20, 22), organize microtubules when they are locally nucleated at a constant rate within a 1 × 1 × 0.2 µm^3^ volume at the center of a simulation box of 30 × 30 × 0.2 µm^3^ (Fig. 1*A*). At steady state, the number of microtubules is determined by the nucleation rate and their lifetime in the bounded growth regime (35) (Fig. 1*B*), while their mean length is determined by the dynamical instability parameters of the microtubule plus ends (*Methods*). We varied the microtubule plus end growth speed in the range from 15 nm/s to 120 nm/s, roughly covering the range of typical speeds of microtubules growing in vitro and getting close to speeds observed in human cells. We varied the nucleation rate and catastrophe frequency to keep the microtubule number at steady state and their mean length of 2.5 µm unchanged (*SI Appendix*, Fig. S1).

Human kinesin-5 was modeled as a symmetric microtubule crosslinker with two processive motor units, both having a speed of 30 nm/s (9, 36, 37) and an unbinding rate of 0.1/s, resulting in an average run length of 300 nm (or 37.5 8-nm steps) (*SI Appendix*, Tables S1 and S2). Importantly, motors at microtubule ends unbind with the same rate as all along the microtubule (i.e., motors dwell at ends determined by their unbinding rate). Kinesin-5 alone generated a bundled microtubule network when the microtubule plus end growth speed was equal to or greater than the motor speed, displaying a nematic organization at higher microtubule growth speeds (*SI Appendix*, Fig. S1*A*). When the motor moved faster than microtubule plus ends grew, kinesin-5 formed an aster with a plus pole at the nucleation source, because the motor could now reach microtubule plus ends and gather them (*SI Appendix*, Fig. S1*A*). This behavior recapitulates what has been observed in the presence of global microtubule nucleation (20), with the difference that here only one network formed around the nucleation source.

Human kinesin-14 HSET was modeled as an asymmetric microtubule crosslinker consisting of one nonprocessive motor unit having a speed of 80 nm/s and an unbinding rate of 5/s, resulting in an average run length of only two 8-nm steps (16 nm) and one diffusive microtubule binding unit with an unbinding rate of 0.01/s (38–40) (*SI Appendix*, Tables S1 and S2). HSET always organized microtubules into asters focusing the nongrowing microtubule minus ends into a single pole at the nucleation source, displaying also a tendency to bundle parallel microtubules (*SI Appendix*, Fig. S1*B*), again replicating what has been observed in systems with global microtubule nucleation (20). Although the HSET motor unit is nonprocessive, HSET can still organize microtubules given the long dwell time of its diffusive binding unit (20, 23).

### Mixtures of kinesin-5 and HSET Fail to Form a Bipolar Spindle Network.

Next, we studied mixtures of these two motors. At equal numbers of plus and minus motors, with identical microtubule growth and kinesin-5 speed, a network formed around the nucleation source with kinesin-5 being more concentrated in the center, whereas HSET clustered minus ends in the network periphery into several poles whose number varied over time (Fig. 1*D*). Microtubule minus ends were distributed further outward than plus ends, demonstrating that under these conditions kinesin-5 wins the competition for antiparallel sliding (*SI Appendix*, Fig. S2 *A*–*C*), as expected from previous experiments and simulations of systems with global microtubule nucleation (22). Computing the number of minus poles from the ratio of minus to plus end densities (*SI Appendix*, Fig. S2*D* and *Methods*) showed considerable pole number fluctuation over time (Fig. 1 *E* and *F*) and rather few microtubule minus ends per pole at a given time (12 ends on average). Analyzing the topology of minus motor links present in the network, we found that less than 1% of all microtubule-to-microtubule links formed by HSET were involved in connecting two minus ends, reflecting the poor pole focusing by HSET in the presence of the plus-motor kinesin-5. Increasing the HSET number while keeping the kinesin-5 number constant, generated unstable networks with higher and still fluctuating numbers of poorly focused poles (Fig. 1*F*). Reducing the HSET number reduced the average pole number and further increased the pole number fluctuations (Fig. 1*F*). Previously, it was concluded for a system with global microtubule nucleation that mixtures of kinesin-5 and HSET are not able to generate networks in which well-focused poles and central extensile bundles coexist (22). These two types of motors do also not organize a stable bipolar network with well-focused poles around a microtubule nucleating source.

### Kinesin-5 and a Symmetric Minus Motor Can Form a Bipolar Spindle via a Monopolar Intermediate.

**Fig. 2.**
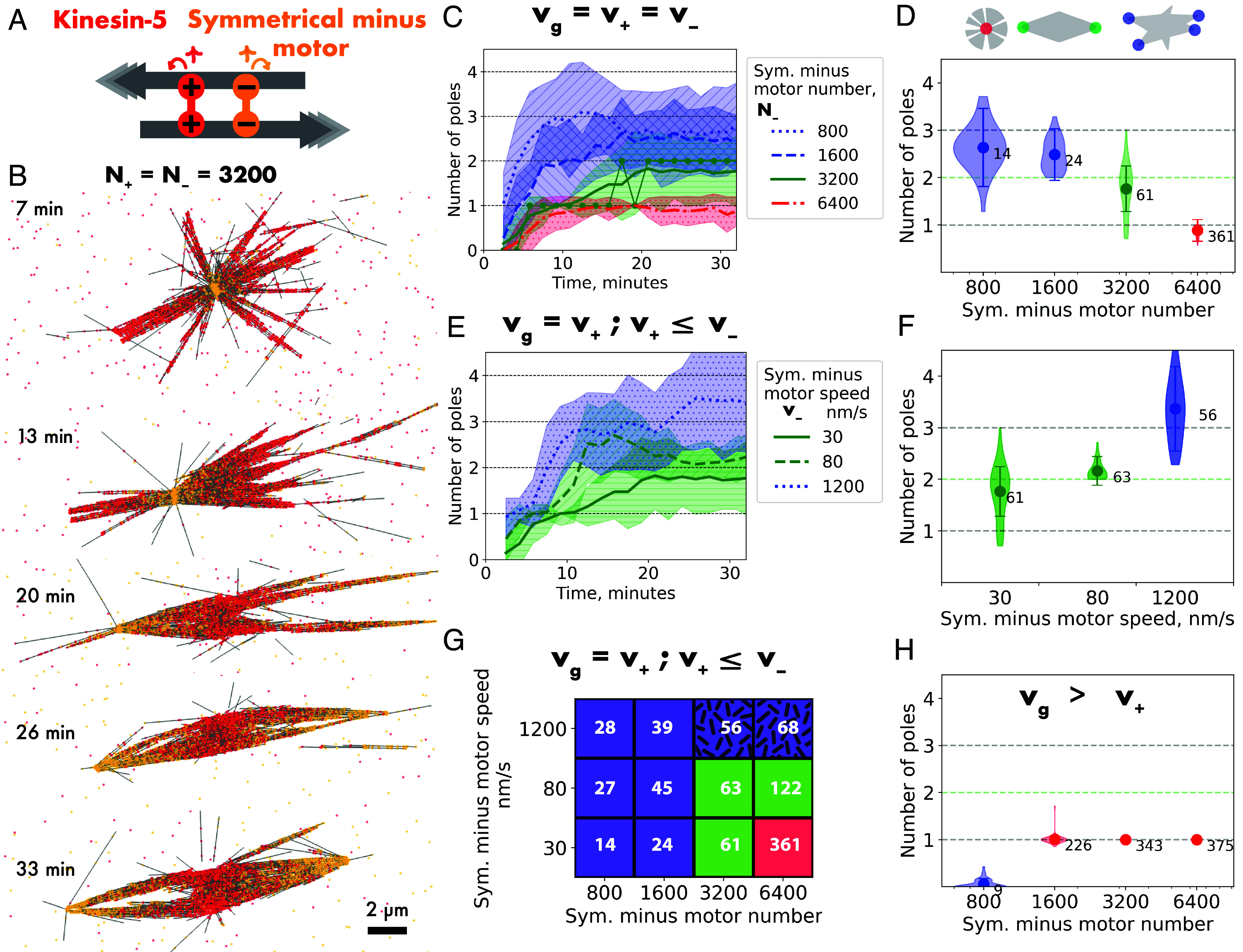
Kinesin-5 and a symmetrical minus motor can organize bipolar spindles via a monopolar intermediate. (*A*) Schematic of plus motor kinsin-5 (red) and a symmetrical minus motor (yellow). Both motors consist of two motor units (symbol + or –) with speed v_+_ = v_–_ = 30 nm/s, but have opposite directionality, and an unbinding rate k_off+_ = k_off−_ = 0.1/s. (*B*) Images of a simulated microtubule network organized by N_+_ = 3,200 kinesin-5 and N_−_ = 3,200 symmetrical minus motors at the indicated times. Microtubule dynamics as in Fig. 1 (growth speed v_g_ = 30 nm/s). (*C*) Time series of the average number of detected poles and (*D*) steady-state pole statistics for simulated networks with different symmetrical minus motor numbers: N_+_ = 3,200, N_−_ = 800 to 6,400, v_+_ = v_–_ = v_g_ = 30 nm/s. Connected green dots in (*C*) correspond to the simulation shown in (*B*). (*E*) Time series of the average number of detected poles and (*F*) steady-state pole statistics for simulated networks with different symmetrical minus motor speeds: N_+_ = N_−_ = 3,200, v_+_ = v_g_ = 30 nm/s, v_–_ = 30 to 1,200 nm/s. (*G*) Phase diagram of microtubule network organization at various combinations of symmetrical minus motor number and speed, including the conditions shown in (*E*) and (*F*). The observed states are color-coded as indicated and the numbers in the grid indicate the number of microtubule minus ends per pole. (*H*) Symmetrical minus motor number variation at increased growth speed: N_+_ = 3,200, N_−_ = 800 to 6,400, v_+_ = v_–_ = 30 nm/s, v_g_ = 120 nm/s. For all conditions, 40 simulations were performed. The means, SD, and microtubule numbers per pole are displayed as in Fig. 1 *E* and *F*. Network organization is color-coded: bipolar (green), multipolar or unfocused (both blue), or monopolar (red). The green data shown in different graphs for simulations with N_+_ = N_−_ = 3,200, v_+_ = v_–_ = v_g_ = 30 nm/s are from the same set of simulations. For a summary of parameters, see *SI Appendix*, Table S2.

**Fig. 3.**
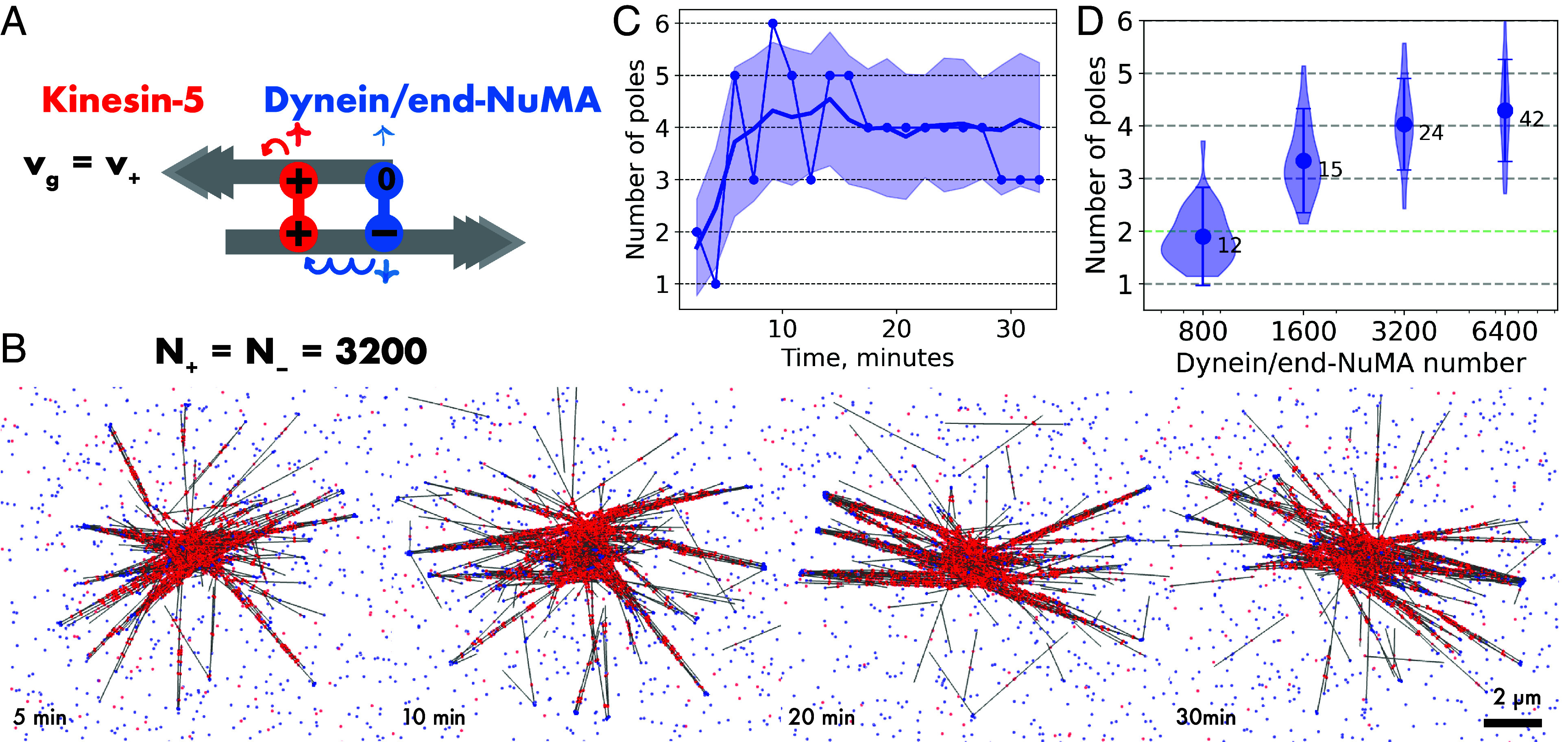
Kinesin-5 and dynein/end-NuMA fail to organize bipolar spindles. (*A*) Schematic of plus motor kinesin-5 (red, with properties as in previous figures) and a minus motor (blue) representing a dynein/NuMA complex with NuMA being assumed to bind only to microtubule minus ends. “Dynein/end-NuMA” consists of a minus motor unit (symbol –, v_–_ = 1.2 µm/s, k_off–_ = 0.1/s) and a static microtubule minus end binding unit (symbol 0, unbinding rate k_off0_ = 0.01/s). Microtubule dynamics as in Fig. 1 (growth speed v_g_ = 30 nm/s). (*B*) Images of a simulated microtubule network with N_+_ = N_–_ = 3,200 kinesin-5 and dynein/end-NuMA motors at the indicated times. (*C*) Time series showing the number of poles (connected dots) for the network in (*B*), and the average number of poles (solid line) and the SD (shaded region) from 40 simulations. (*D*) Steady-state pole statistics of various networks with N_+_ = 3,200 kinesin-5 motors and N_–_ = 800 to 6,400 dynein/end-NuMA motors, displayed as in previous figures. For a summary of parameters, see *SI Appendix*, Table S2.

**Fig. 4.**
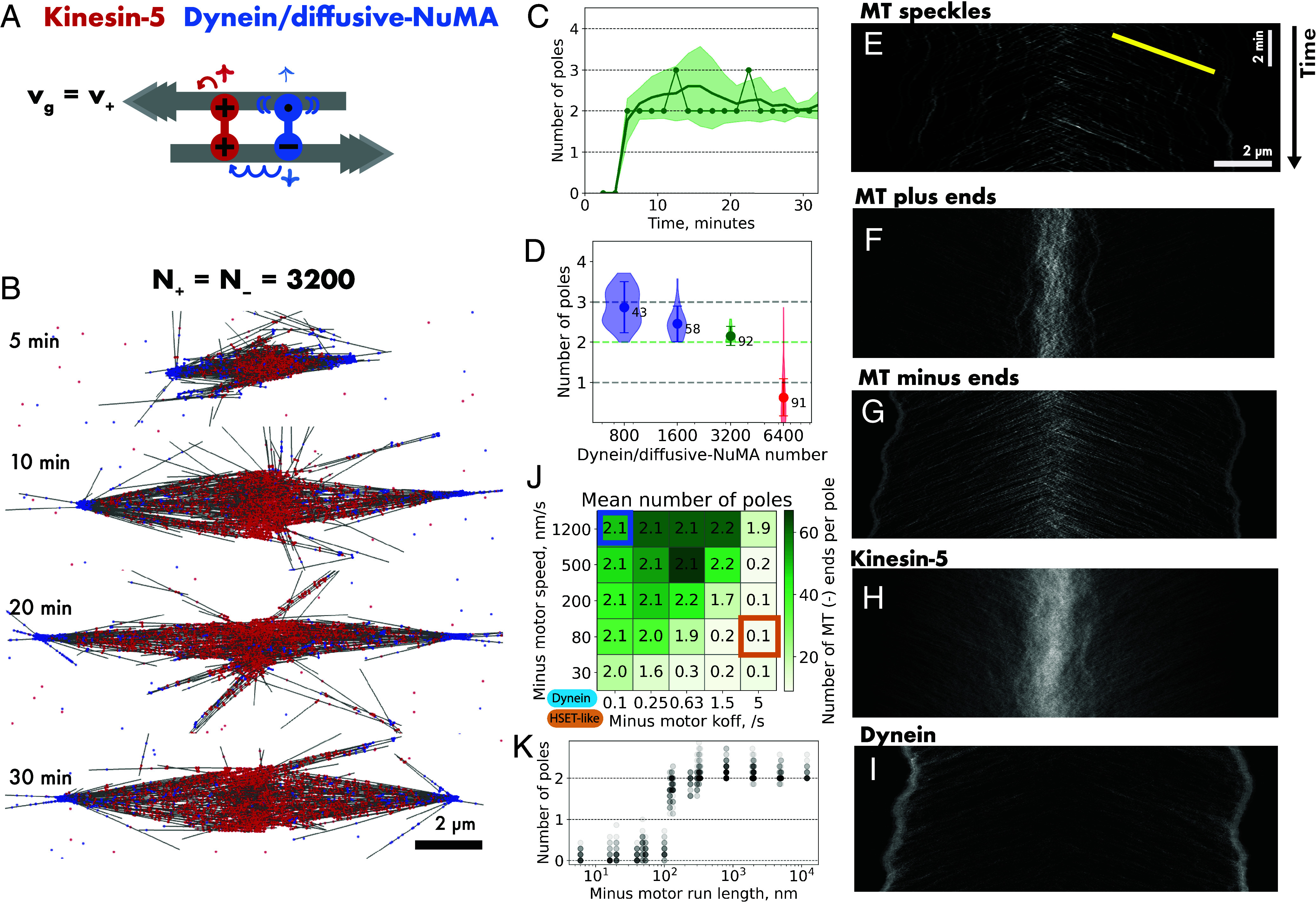
Kinesin-5 and dynein/diffusive-NuMA robustly organize bipolar spindles. (*A*) Schematic of plus motor kinesin-5 (red) and a minus motor (blue) representing a dynein/NuMA complex with NuMA being assumed to bind and diffuse all along a microtubule. “Dynein/diffusive-NuMA” consists of a minus motor unit (symbol –, v_–_ = 120 nm/s, unbinding rate k_off–_ = 0.1 /s), and a diffusive microtubule binding unit (symbol •, diffusion coefficient = 0.1 µm^2^/s, unbinding rate k_offd_ = 0.01/s). Microtubule dynamics as in Fig. 1 (growth speed v_g_ = 30 nm/s, N_MT_ = ~ 358, L_MT_ = 2.5 µm, τ_MT_ = ~ 1.5 min). (*B*) Images of a simulated self-organizing microtubule network with N_+_ = N_–_ = 3,200 kinesin-5 and dynein/diffusive NuMA motors. (*C*) Time series showing the number of poles (connected dots) in the network shown in (*B*), and the mean number of poles (solid line) and the SD (shaded region) from 40 simulations. (*D*) Steady-state pole statistics of networks with N_+_ = 3,200 kinesin-5 and N_–_ = 800 to 6,400 dynein/diffusive-NuMA motors, displayed as in previous figures (for color code, see Fig. 2). (*E*–*I*) Kymographs showing movements along the spindle axis of (*E*) randomly positioned “speckles” on microtubules (the yellow line shows the slope of 30 nm/s), (*F*) microtubule plus ends, (*G*) microtubule minus ends, (*H*) kinesin-5 motors, and (*I*) dynein/diffusive-NuMA motors in the network shown in (*B*) during the last minutes of the simulation (25 to 33 min). (*J*) Phase space of microtubule network organization with kinesin-5 motors and minus motors consisting of one motor and one diffusive binding unit, varying the minus motor speed v_-_ and unbinding rate k_off−_. Grid numbers represent the mean of the number of poles from 30 simulations, the color shade indicates the mean number of microtubule minus ends per pole. Blue and orange frames indicate simulations with dynein/diffusive-NuMA and HSET motor speed and unbinding properties. The minus motor binding rate, however, is here 0.1/s, different from that of the HSET model in Fig. 1 and *SI Appendix*, Figs. S1 and S2 and Table S1). (*K*) Mean pole number plotted as function of minus motor run length (L_–_ = v_−_/k_off−_) for all simulations in (*J*). Dot intensity increases with numbers of simulations contributing to the dot value. For a summary of parameters, see *SI Appendix*, Table S2.

**Fig. 5.**
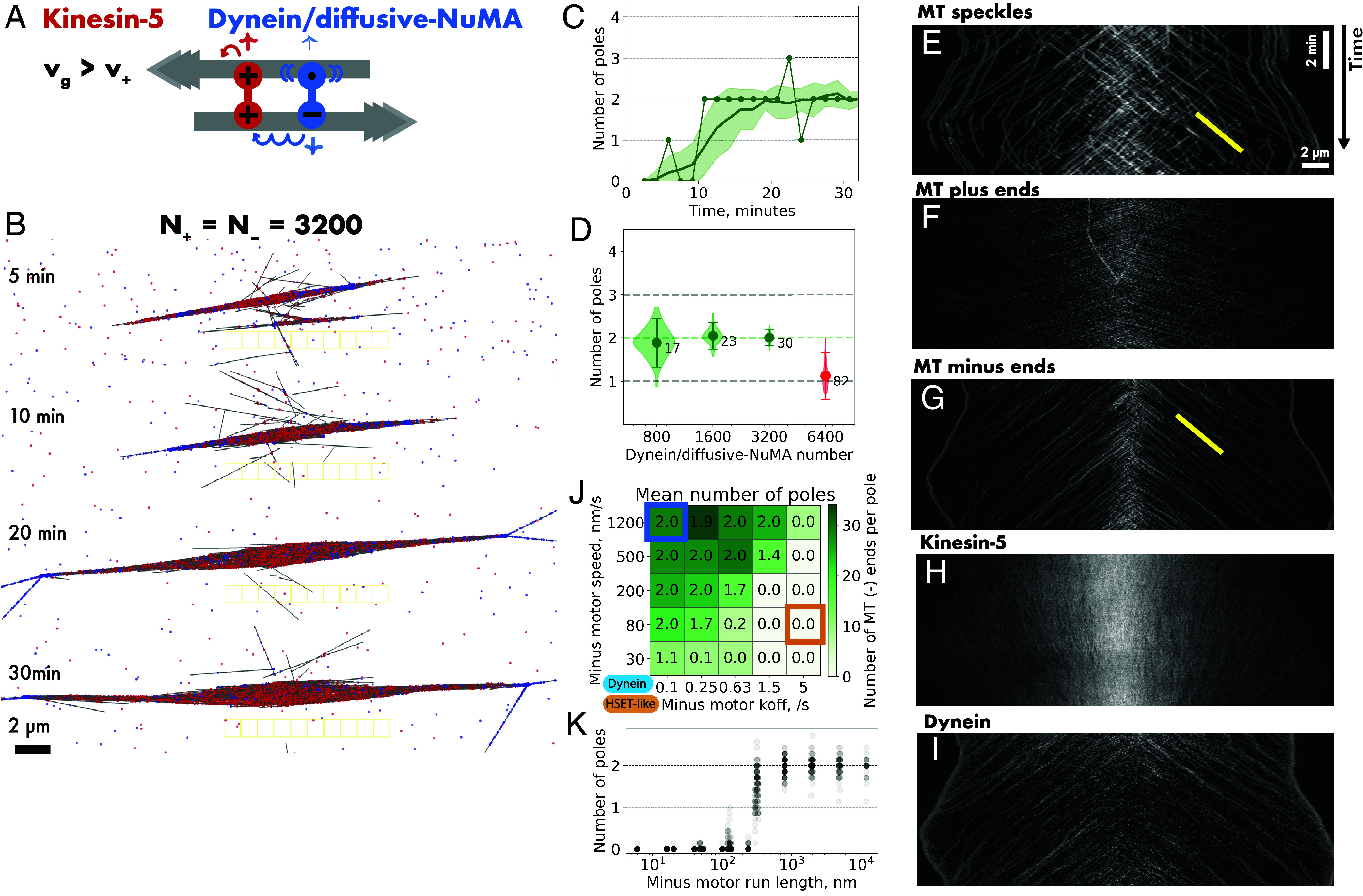
Kinesin-5 and dynein/diffusive-NuMA can organize bipolar spindles also at faster microtubule growth. (*A*) Schematic of a kinesin-5 (red) and dynein/diffusive NuMA (blue) with properties as described for Fig. 4*A*. Microtubules grow now with 4× increased speed, nucleating at an adjusted rate to keep the total microtubule mass as in Fig. 4 (v_g_ = 120 nm/s, N_MT_ ~ 179, L_MT_ = 5 µm, τ_MT_ = ~ 1.6 min). (*B*) Images of a simulated self-organizing microtubule network with N_+_ = N_–_ = 3,200 kinesin-5 and dynein/diffusive-NuMA motors. (*C*) Time series showing the number of poles (connected dots) in the network shown in (*B*), and the mean number of poles (solid line) and the SD (shaded region) from 40 simulations. (*D*) Steady-state pole statistics of networks with N_+_ = 3,200 kinesin-5 and N_–_ = 800 to 6,400 dynein/diffusive-NuMA motors, displayed as in previous figures (for color code, see Fig. 2). (*E*–*I*) Kymographs showing movements, as indicated, along the axis of the spindle in (*B*) during the last minutes of the simulation (25 to 33 min). (*J*) Phase space of microtubule network organization with kinesin-5 motors and minus motors consisting of one motor and one diffusive binding unit, varying the minus motor speed v_−_ and unbinding rate k_off−_. Grid numbers represent the mean number of poles from 30 simulations, the color shade indicates the mean number of microtubule minus ends per pole. Blue and orange frames indicate simulations with dynein/diffusive-NuMA and HSET motor speed and unbinding properties. The minus motor binding rate, however, is here 0.1/s, different from that of the HSET model in Fig. 1 and *SI Appendix*, Figs. S1 and S2 and Table S1. (*K*) Mean pole number plotted as function of minus motor run length (L_–_ = v_−_/k_off−_) for all simulations in (*J*). Dot intensity increases with numbers of simulations contributing to the dot value. For a summary of parameters, see *SI Appendix*, Table S2. In contrast to previous figures, simulations were performed in a container measuring 50 × 50 × 0.2 µm^3^.

**Fig. 6.**
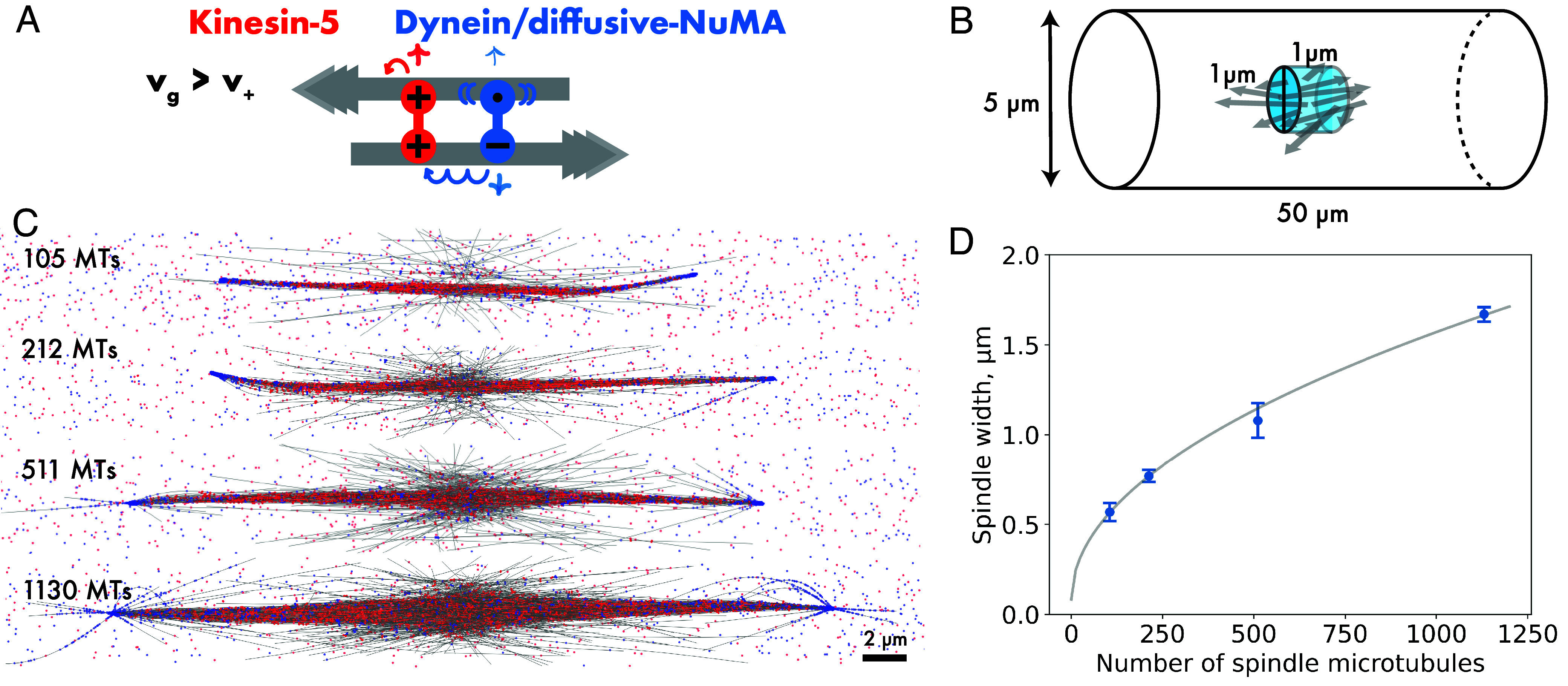
Kinesin-5 and dynein/diffusive-NuMA also organize bipolar spindles in a cylindrical geometry. (*A*) Schematic of a kinesin-5 (red) and dynein/diffusive-NuMA (blue) with properties as described for Figs. 4*A* and 5*A* and microtubule dynamics as in Fig. 5 (fast growth with v_g_ = 120 nm/s). (*B*) Schematic of the 3D simulation space with reflective boundaries. Not to scale. (*C*) Images of simulated microtubule networks with different mean microtubule numbers at steady state, as indicated, organized by kinesin-5 and dynein/diffusive-NuMA after 33 min. Microtubule nucleation rate = 2, 4, 8, 16/s and motor numbers N_+_ = N_−_ = 3,200, 4,800, 9,600, and 19,000, resulting in a ratio of crosslinking motors to spindle microtubules of ~19 for all simulations. (*D*) Spindle width as a function of spindle microtubule number from 10 simulations each. Error bars are SD, the line is a square root function fit.

**Fig. 7.**
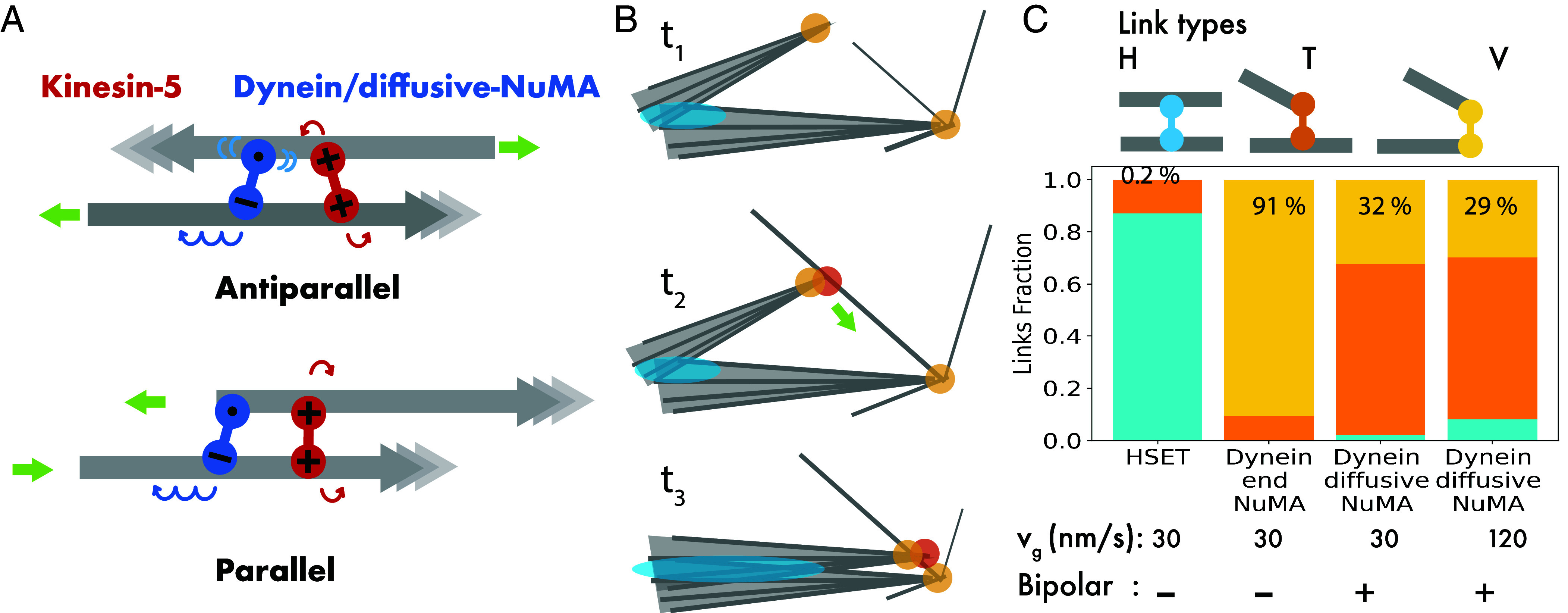
Design of motor crosslinks that promote bipolar spindle network organization. (*A*) Schematic showing plus motor kinesin-5 (red) and minus motor dynein/diffusive-NuMA (blue) crosslinking an antiparallel (*Top*) and parallel (*Bottom*) pair of microtubules. (*B*) Schematic sequence illustrating lateral pole fusion, mediated by minus motors engaged in end-to-side microtubule connections (T-links). (*C*) *Top*: Link types: H-link (cyan, side-to-side microtubule connection), T-link (orange, end-to-side connection), and V-link (yellow, end-to-end connection). *Bottom*: The fraction of links formed in networks with kinesin-5 and different types of minus motors, kinesin-14 HSET, dynein/end-NuMA, dynein/diffusive-NuMA, for growth speeds v_g_, as indicated.

We then redesigned the minus motor attempting to improve its pole focusing activity in the presence of kinesin-5. We modeled it as a motor with kinesin-5 properties, but with minus end directionality, i.e., as an unnatural symmetric minus-directed crosslinker consisting of two processive motor units (Fig. 2*A*). When kinesin-5 and this symmetric minus motor were present in equal numbers, the minus motor dominated initially, generating an aster with a minus pole (Fig. 2*B*—7 min, *SI Appendix*, Fig. S3*A*). This is because the processive symmetric minus motor competes more efficiently with kinesin-5 than the nonprocessive HSET motor with its diffusive unit. Because the processive minus motor can reach more easily the nongrowing microtubule minus ends than kinesin-5 the growing plus ends, it quickly connects microtubule minus ends (representing about 31% of all minus motor links), leading to the formation of an aster.

Remarkably, however, the aster was unstable. After some time, a second minus pole developed and became separated from the first pole, with a kinesin-5-rich region in between. This process was supported by constant local microtubule nucleation and turnover and by antiparallel kinesin-5-driven sliding of microtubules away from the first pole after an initial symmetry-breaking event. Finally, a bipolar spindle network with two focused poles formed around the nucleation source, establishing a new network symmetry (Fig. 2*B*—26 min, *SI Appendix*, Fig. S3*B*). Once the bipolar network was organized, it was stable over time (Fig. 2*C*—green) and the number of minus ends in the two poles at a given time was high (61 ends per pole on average, Fig. 2*D*—green), demonstrating good pole focusing.

### Bipolar Spindle Formation by Two Symmetric Motors of Opposite Directionality Requires Tightly Balanced Conditions.

Next, we tested how sensitive bipolar spindle formation is to the number of symmetric minus motors (Movie S1). With fewer minus motors than kinesin-5 motors, the plus motor dominated from the start, preventing initial aster formation, eventually leading to mostly multipolar networks with poorly focused poles and fluctuating pole numbers (Fig. 2 *C* and *D*—blue). Apparently, microtubule sliding by the now dominant plus motor interfered with minus pole focusing. In contrast, with twice as many minus motors than kinesin-5 motors, a stable aster formed with a minus pole that persisted over time (Fig. 2 *C* and *D*—red). The minus motor was now apparently too dominant, preventing kinesin-5-driven symmetry breaking needed for bipolar spindle formation.

Next, we increased the speed of the symmetric minus motor, keeping first plus and minus motor numbers equal. A mild increase to 80 nm/s (speed of the HSET motor) also supported the robust formation of stable bipolar spindles with well-focused poles after initial monopolar aster formation (Fig. 2 *E* and *F*—green, 80 nm/s). Spindles were even a little more stable and the number of minus ends per pole even slightly higher compared to the slower minus motor. Increasing the speed further to 1.2 µm/s (speed of the dynein motor) again led to the initial formation of a monopolar aster followed by pole splitting, but networks now became multipolar with fairly well-focused poles, but fluctuating pole numbers (Fig. 2 *E* and *F*—blue). In this case, 96% of all minus motor links were now involved in connecting minus ends, leaving only few minus motors bound to the side of microtubules where they can contribute to fuse adjacent poles together. This result shows that if the minus motor is too efficient in reaching microtubules minus ends, multipolarity can arise.

Looking at a larger section of the phase space of network organization in the presence of kinesin-5 and a symmetric minus motor, we can classify four types of emergent organization (Fig. 2*G*): i) When there are fewer minus motors than kinesin-5 motors, the plus motors dominate and multipolar networks form with poorly focused poles (Fig. 2*G*—plain blue). When kinesin-5 does not dominate, ii) monopolar asters form when the minus motor is slow and in excess (no monopole splitting, Fig. 2*G*—red), iii) multipolar spindles form when the minus motor is fast (monopole splitting occurs but parallel bundling is poor, Fig. 2*G*—blue textured), or iv) at an intermediate regime between (ii) and (iii) bipolar spindles assemble (pole splitting occurs, both pole focusing and parallel bundling are well supported by the minus motors, Fig. 2*G*—green).

### Bipolar Spindles Cannot Be Generated by Two Symmetric Motors of Opposite Directionality When Microtubule Growth and Plus Motor Speed Do Not Match.

Next, we tested how the microtubule growth speed affects network organization by equal numbers of the two symmetric motors with opposite directionality and same speed. Decreasing the microtubule growth speed to half the kinesin-5 speed and adjusting dynamic instability parameters such that the average microtubule length remained the same led to the formation of a new type of network with one plus and one minus pole connected by a parallel microtubule array (*SI Appendix*, Fig. S4*B*). Both motors formed poles individually, given that kinesin-5 can now also reach microtubule plus ends at this reduced plus end growth speed (*SI Appendix*, Fig. S1*A*). This type of organization represents the isolated unit of a previously experimentally observed network of alternating poles generated by two artificial motor oligomers with opposite directionality that were able to reach their respective microtubule ends under conditions of global microtubule nucleation (21).

Increasing or decreasing the number of minus motors at the decreased microtubule growth speed led to the formation of asters with either a single minus or single plus pole, respectively (*SI Appendix*, Fig. S4 *A* and *C*), depending on which motor dominated. Bipolar spindles with two minus poles did not form when microtubules grew so slowly.

Conversely, when microtubules grew 4 times faster than kinesin-5 walks, adjusting the nucleation rate and dynamic instability parameters such that microtubules now had a mean length of 5 µm, asters with a minus pole formed when the motor numbers were balanced (Fig. 2*H* and *SI Appendix*, Fig. S4*D*), and also when the minus motor number was varied by a factor of two (Fig. 2*H*). Asters did not develop into bipolar spindles, because antiparallel microtubules could not be moved away from the pole by kinesin-5 as their plus ends grew over the minus pole and they were pulled by pole-accumulated minus motors toward the pole until their minus ends ended up at the pole, becoming part of the aster. Decreasing the number of minus motors did not solve this issue, because then the network failed to consistently form poles (Fig. 2*H*). A larger section of the phase space of network organization, varying microtubule growth speed and minus motor speed, confirms that the region of bipolar spindle formation is small (*SI Appendix*, Fig. S4*E*).

In conclusion, two symmetric processive motors with opposite directionality can organize asymmetrically growing microtubules into bipolar spindles via an unstable monopolar intermediate, but only within a very limited parameter regime. Motor numbers and speeds need to be balanced and microtubule growth speed must match plus motor speed. This scenario is very different from the situation in cells where microtubules typically grow much faster than the speed of kinesin-5 (41–43).

### A Dynein/NuMA-Like Motor with a Microtubule Minus End Binding Unit Cannot Organize Bipolar Spindles with Kinesin-5.

In animal cells, the major spindle pole focusing motor is dynein (4, 10, 44), which, however, requires NuMA for pole focusing (10, 14, 16). NuMA can directly bind dynein and has been shown to localize to microtubule minus ends in cells (15, 16, 45). Therefore, we tested a model with a dynein/NuMA-like microtubule crosslinker made of one fast and processive minus motor unit (1.2 µm/s speed, 0.1/s unbinding rate), representing dynein, and one unit that binds only to microtubule minus ends (0.01/s unbinding rate), representing NuMA with minus end binding specificity (dynein/end-NuMA).

Equal numbers of kinesin-5 and dynein/end-NuMA generated a multipolar network with a fluctuating pole number (Fig. 3 *B* and *C* and *SI Appendix*, Fig. S5*A*). With fewer dynein/end-NuMA motors the quality of pole focusing decreased as indicated by the reduced number of microtubules per pole (Fig. 3*D*). Adding more dynein/end-NuMA motors increased the number of microtubules per pole, but networks were still multipolar (Fig. 3*D*). Like the fast symmetrical minus motor, dynein/end-NuMA focused microtubule minus ends too efficiently, as indicated by a large number of crosslinks holding minus ends together (>90% of all minus motor crosslinks), at the expense of other links that are needed to keep the poles on either side of a spindle network clustered together.

These results differ from the behavior of a previous one-dimensional “slide and cluster” model of spindle organization by two opposite-directionality motors where dynein was also modeled as a motor that specifically binds to microtubule minus ends (27). It was additionally assumed that dynein could connect only parallel microtubules. Adding this property to our dynein/end-NuMA model did however not produce stable bipolar spindles (*SI Appendix*, Fig. S6 *A*–*C*). Only when the network was spatially constrained to assemble in a narrow channel, microtubules became organized into a stable bipolar spindle (*SI Appendix*, Fig. S6*D*). This result shows how reducing spatial dimensionality can solve the problem of too efficient minus end accumulation of this minus motor model which however causes spindle multipolarity in higher dimensional space, failing to generate bipolar spindles.

### A Dynein/NuMA Crosslinker with a Diffusive Microtubule Binding Unit Can Robustly Generate Bipolar Spindles with Kinesin-5.

Because purified NuMA has been shown to diffuse along microtubules in vitro (46), we modified our dynein/NuMA model, combining a dynein-like motor with a microtubule binder that can bind and diffuse all along a microtubule and unbind with the same slow unbinding rate as the diffusive unit of the HSET model (dynein/diffusive-NuMA) (*SI Appendix*, Table S1). Remarkably, this type of active microtubule crosslinker readily organized microtubules into bipolar spindles together with kinesin-5 at equal plus and minus motor numbers (Fig. 4*B*, *SI Appendix*, Fig. S7*A*, and Movie S2). Two spindle poles developed simultaneously (Fig. 4*C*), very different from the pathway of spindle formation with the hypothetical symmetric minus motor.

Reducing the number of dynein/diffusive-NuMA crosslinkers resulted in networks with an unstable number of poles, often displaying multipolar structures, and reduced the number of microtubules per pole (Fig. 4*D*), indicative of the plus motor now being too dominant, as observed also for the other tested minus motors before when their numbers were low. Increasing the number of dynein/diffusive NuMA led to the formation of asters with a single minus pole, because now the minus motor was too dominant.

To visualize the internal dynamics of the spindles formed by equal numbers of motors, we generated kymographs of randomly placed labels (“speckles”) along the microtubules, which showed that microtubules were transported to the two poles roughly at the kinesin-5 speed of ~30 nm/s (Fig. 4*E* and Movie S3), revealing little resistance to kinesin-5-driven antiparallel microtubule sliding from dynein/diffusive NuMA, similar to the situation in cells (47). While microtubule plus ends were enriched in the spindle center (Fig. 4*F*), minus ends were transported to the poles where they accumulated (Fig. 4*G*). In the spindle center, kinesin-5 appeared mostly static as it slid microtubules outward (Fig. 4*H*), whereas dynein/diffusive-NuMA became enriched at spindle poles (Fig. 4*I*), however not too excessively; only 32% of all minus motor crosslinks were involved in holding minus ends together, so that sufficient crosslinks remained available to stabilize half-spindles throughout as indicated by 66% minus motor crosslinks connecting minus ends to the sides of parallel microtubules. This ensures spindle bipolarity by efficient minus end focusing and pole clustering on either side of the spindle network via sufficient parallel microtubule interactions.

We have also tested a dynein/diffusive NuMA model with slower motor speeds, while maintaining a high processivity. We found that bipolar spindles were still generated in a similar motor number range as for the faster dynein/diffusive-NuMA model (*SI Appendix*, Fig. S7 *B* and *C*), suggesting that high minus motor processivity and not necessarily speed is important to support bipolar spindle formation. To further validate this, we explored a wider range of minus motor speeds and unbinding rates at equal plus and minus motor numbers. The resulting phase space reveals a broad parameter region where bipolar spindles form (Fig. 4*J*) and indeed identifies the run length of the minus motor as a critical control parameter (Fig. 4*K*).

### Dynein/Diffusive-NuMA and Kinesin-5 Generate Bipolar Spindles Also When Microtubule Plus Ends Grow Fast.

Next, we increased the microtubule growth speed fourfold and found that bipolar spindles with two stable, well-focused poles still formed at balanced motor numbers (Fig. 5 *B* and *C*, *SI Appendix*, Fig. S8*A*, and Movie S4), in marked contrast to the networks formed by two symmetrical motors. Spindles still formed even when the microtubules grow much faster than kinesin-5 walks, because spindle formation does not proceed via an aster intermediate from which a second pole needs to emerge. In the presence of dynein/diffusive-NuMA both poles formed simultaneously, also at these elevated microtubule growth speeds (Fig. 5 *B* and *C*). Spindles were longer and thinner compared to the slower growth speed, because microtubules were longer, but also fewer, as we had reduced the nucleation rate to keep the total polymerized microtubule mass constant.

Increasing the dynein/diffusive-NuMA number caused the formation of monopolar asters (Fig. 5*D*), as in the simulations with slower microtubule growth. With fewer dynein/diffusive-NuMA than kinesin-5, bipolar spindles still formed, even if fewer microtubule minus ends were incorporated in the poles and the number of poles was less stable compared to the condition with balanced plus and minus motor numbers (Fig. 5*D*). These results show that at higher growth speed bipolarity can be obtained over a larger range of motor ratios (Movie S5).

Kymographs showed similar internal spindle dynamics as for the slower microtubule growth speeds (Fig. 5 *E*–*I* and Movie S6). But microtubule plus ends now grew visibly faster, forming larger central spindle microtubule overlaps (Fig. 5*F*) with kinesin-5 being enriched in these overlaps over a wider region (Fig. 5*H*), suggesting an explanation why bipolarity can be obtained in a more robust manner at faster microtubule growth.

Again, bipolar spindles formed in a wide regime of minus motor speeds and unbinding rates (Fig. 5*J*), with the motor run length being an important control parameter (Fig. 5*K*).

Finally, to investigate the potential impact of our thin (0.2 µm high) simulation space on spindle organization in the presence of kinesin-5 and dynein/diffusive NuMA (Fig. 6*A*), we performed additional simulations with fast-growing microtubules in a cylinder of 50 µm length and 5 µm diameter (Fig. 6*B*). In this volume, spindles oriented themselves along the long axis, and were now much less spatially constrained. Bipolar spindles still formed robustly but were thinner, indicating that the flat box had compressed the spindle, causing it to widen in one dimension. Spindles in the cylindrical space became wider when the number of microtubules was increased, keeping the number of spindle-bound motors per number of microtubules roughly constant (Fig. 6*C* and Movie S7). Spindle width varied with the square root of the spindle microtubule number, corresponding to the surface of the cross-sectional spindle area scaling linearly with the number of microtubules (Fig. 6*D*).

These simulations demonstrate that a symmetrical plus motor and an asymmetrical minus motor with a processive motor unit and a diffusive nonmotor unit, resembling dynein/NuMA in cells, are sufficient to robustly generate bipolar spindles around a local nucleation source of dynamic microtubules in three-dimensional space.

## Discussion

Using stochastic computer simulations, we demonstrated that bipolar spindle-like networks can emerge from a surprisingly small set of active components, suggesting that dynamic microtubules and two motors of opposite directionality represent the core components of a spindle. By varying critical motor properties—difficult or impossible to achieve experimentally—we found that distinct motor designs are critical for bipolar network formation, suggesting an explanation why kinesin-5 and dynein are the two main spindle motors in many cells.

To complement kinesin-5, we found that the minus-motor should consist of a processive motor unit and a diffusive microtubule binding unit that dwells at minus ends for some time, in line with the measured characteristics of a dynein/NuMA complex (46, 48). This may explain why dynein is the main pole focusing motor in animal cells and why NuMA is also indispensable for pole focusing. In the future, it will be important to clarify experimentally how kinesin-5 and dynein/NuMA organize microtubules together, ideally through in vitro reconstitution experiments.

Dynein/diffusive-NuMA and sufficiently processive variants were the only type of minus motor that supported robust bipolar spindle formation together with kinesin-5 at microtubule growth speeds similar to those observed in animal cells. A slow and nonprocessive motor with a separate diffusive microtubule-binding domain, representing human kinesin-14 HSET did not support stable bipolar spindle formation together with kinesin-5, in agreement with previous, more complex spindle simulations of unfocused meiotic spindles (28). These results may explain why HSET normally plays only a supporting role for pole focusing in animal cells (44).

Kinesin-5 is well suited to organize microtubules into nematic arrays of extensile bundles unless microtubules grow extremely slowly (20). Our simulations explain why a crosslinker with dynein/diffusive-NuMA properties is the ideal partner for kinesin-5 to organize microtubules into bipolar spindles. First, this minus-directed crosslinker design avoids a direct tug-of-war between dynein and kinesin-5 when they connect antiparallel microtubules. Kinesin-5 can slide microtubules apart and produce poleward flux, because the diffusive NuMA unit produces only a weak counterforce (Fig. 7 *A*, *Top*). However, when connecting parallel microtubules, the fast and processive dynein will be able to pull a minus end toward another minus ends, because NuMA remains bound for some time upon reaching a minus-end. Therefore, dynein-produced forces will be exerted preferentially in regions with parallel microtubules specifically at minus ends, contributing to the formation of poles (Fig. 7 *A*, *Bottom*).

The design of dynein/diffusive-NuMA also prevents excessive accumulation of minus-directed crosslinkers at microtubule minus ends which can cause multipolarity due to failure of lateral pole clustering when multiple poles form on either side of the network (Fig. 7*B*). This can be seen by examining the topological crosslink types that the different minus-motors form in different networks (Fig. 7 *C*, *Top*). At steady state, bipolar spindle-like networks formed by kinesin-5 and dynein/diffusive-NuMA have mostly microtubule side-to-end (T) links, followed by end-to-end (V) links (Fig. 7 *C*, *Bottom*). In contrast, networks with dynein/end-NuMA have many V, but only few T links, making them multipolar despite poles being well-focused. And networks with HSET have mostly microtubule side-to-side (H) links, few T links and hardly any V-links, leading to a network with poorly focused and unstable poles.

A symmetric minus motor with two processive motor units antagonizes antiparallel kinesin-5 driven microtubule sliding more efficiently than a minus motor with a diffusible binding part. As a result, spindle formation is possible only in a very narrow parameter regime, where an aster with a minus pole first forms, followed by kinesin-5-driven sliding of antiparallel microtubules nucleated next to the pole, which are then focused by the minus motor into a second pole. This pathway requires the microtubule growth and kinesin-5 speeds to match, which is not the case in animal cells, which explains why this pathway is not observed in cells.

In our simulations bipolarity emerges when plus and minus motor numbers are approximately balanced. Mass spectrometry data show a roughly threefold to fourfold excess of dynein and NuMA over kinesin-5 in human cells (49). Several reasons may explain this difference in motor ratios between our simulations and cells. Part of dynein and NuMA localizes in cells to the cortex to interact with microtubule plus ends for spindle positioning and is therefore not available for spindle pole organization (50). Dynein is also involved in various other transport processes (51) and protein activities may be regulated in cells. Moreover, in the simulations the binding rates of the motors have been set to reasonable values, as they have not been measured yet, but, adjustment of motor numbers could be required if the binding rates were different from our estimated values, because to a first approximation only bound motors matter for network organization.

Despite the simplicity of our model, we observed robust self-organization of networks consisting of a central antiparallel microtubule array displaying microtubule flux coexisting with two minus poles in the presence of noise. Several asymmetric system features were essential for this to occur: 1) the two microtubule ends have very different dynamic properties, and 2) two opposite-directionality motors have very distinct molecular designs, one being a symmetric and the other an asymmetric, partly slippery crosslinker.

Despite our simple model, simulation parameter space is still highly multidimensional and we explored only a small part of it, making it possible that other spindle solutions may exist. Mean field approaches could be a path in the future to obtain a more comprehensive picture of the behavior of two-motor models of bipolar spindle formation.

In contrast to some previous spindle models (28, 29), our simulations did not contain a microtubule minus end depolymerase thought to be necessary to maintain constant metaphase spindle length in the presence of outward kinesin-5 driven microtubule sliding. Our finding that minus end depolymerization is not necessarily required to organize minimal bipolar spindles agrees with a conclusion from a previous one-dimensional “slide and cluster” model (27). However, to ensure bipolarity in more than one dimension, we show here that the minus motor must cluster microtubule minus ends not only along the spindle axis but also laterally. The design of dynein/diffusive-NuMA allows exactly that, making the previous hypothetical assumption of dynein not crosslinking antiparallel microtubules unnecessary.

The self-organized spindles generated in our simulations are highly dynamic. Poles sometimes split or disappear before bipolarity is reestablished. This emphasizes the stochastic nature of our model and may indicate that other activities are required to enhance spindle stability, such as branched microtubule nucleation, additional supporting motors, centrosomes, kinetochore fibers, or protein condensates at spindles poles, activities that all have been reported in spindles in cells and were not implemented in the simple model studied here. In the future, it will be interesting to investigate to which extent such additional activities may make bipolar spindle formation sufficiently robust so that they can reliably perform their function.

## Methods

### Computational Model.

We simulated active networks consisting of microtubules and motors using Cytosim (https://gitlab.com/f-nedelec/cytosim). In brief, microtubules are modeled as incompressible, flexible, dynamic filaments. The motion of each filament follows an overdamped Langevin equation, influenced by bending elasticity, spring-like steric interactions, crosslinker-mediated forces, and random forces (33).

The dynamics of the microtubule plus end are described by a two-state model: a growth state with a growth speed *v_g_*, and a shrinkage state with a shrinkage speed *v_s_*. Transitions between these states occur with a catastrophe rate, *k_cat_*, and rescue rate, *k_res_*. The steady-state mean microtubule length and lifetime are given by *L* = *v_g_v_s_/(v_s_k_cat_-v_g_k_res_)* and τ = *(v_g_+v_s_)/(v_s_k_cat_-v_g_k_res_)*, respectively. Most simulations were performed in a flat three-dimensional geometry with reflecting boundary conditions in the X, Y, Z dimensions. The size of the simulation box was much larger than the microtubule length, that is L_x_ = L_y_ = 30 µm or 50 µm for mean microtubule lengths L = 2.5 µm or 5 µm, respectively. The height of the box (L_z_) was set to 0.2 µm, providing sufficient space for microtubules to cross each other. Microtubules were introduced at a constant rate with an initial length of 0.01 µm, and random orientation within a 1 × 1 × 0.2 µm^3^ volume at the center of the simulation box. Some simulations were performed in a cylinder of 5 µm diameter and 50 µm length, also with reflecting boundary conditions (Fig. 6). Microtubules were introduced within a central cylindrical volume of 1 µm diameter and 1 µm length. At steady state, the rate of new microtubule appearance (nucleation) balanced the rate at which microtubules disappeared due to complete shrinkage. We set a nucleation rate that resulted in a dense microtubule network offering many possibilities for connections. Each simulation ran for 33 min of real time, as this yielded a stable number of poles in bipolar networks. For visualization, the three-dimensional networks are shown as two-dimensional projections along the short axis of the simulation space. Given that the simulations are stochastic, we performed 30 or 40 simulations for each condition, as indicated in the legends, to allow statistical analysis.

Microtubule crosslinkers diffusing in the medium can stochastically bind to one or two microtubules through their two microtubule binding units. The binding unit detaches from the microtubule at a force-dependent unbinding rate, $k=k_{\mathrm{off}}e^{\left|f_{\mathrm{load}}\right|/f_{\mathrm{unbind}}}$. A crosslinker that connects two microtubules forms a Hookean spring, characterized by a rest length and a spring constant. There is no angular constraint on the link’s attachment point to the microtubule, allowing the two connected microtubules to rotate freely. A bound molecular motor unit travels along the microtubule with a speed decreasing linearly with the impeding load: $v=v_{m}\left(1+\vec{f_{\mathrm{load}}}\cdot \vec{d}/f_{\mathrm{stall}}\right)$, where $v_{m}$ is the unloaded motor speed, as mentioned in the text, $\vec{f_{\mathrm{load}}}$ is the force vector, $\vec{d}$ is the unit vector along the microtubule in the direction of the motor movement, and $f_{\mathrm{stall}}$ is the motor’s stall force. When a motor reaches the end of a microtubule, it unbinds with its unbinding rate, k. A bound diffusive microtubule-binding unit can diffuse passively along the microtubule lattice in a one-dimensional manner (with a hopping rate = *D/a*^2^, where *D* is diffusion constant and *a* is lattice size) or it can undergo biased diffusion when under load, with an effective speed = *DΔU/ak_B_T*, where *ΔU* is the energy difference between forward and backward steps (52, 53). This diffusive unit detaches with its unbinding rate k at the side and end of a microtubule.

Kinesin-5 is modeled as a symmetrical crosslinker consisting of two processive plus-end directed motor units. The properties of the motor are set to mimic those of kinesin-5. For the minus motor, we tested several different designs, representing the kinesin-14 HSET, an unnatural symmetrical motor, and two types of dynein/NuMA complexes. HSET consists of a nonprocessive minus-end-directed motor unit and a diffusible microtubule binding unit, as implemented previously (22). The symmetrical minus motor is similar to kinesin-5 but has two minus-end directed motor units. Dynein/end-NuMA is composed of a processive minus-end directed motor unit and a microtubule binding unit that binds exclusively to microtubule minus ends. In some simulations dynein/end-NuMA can only crosslink microtubules in a parallel, but not antiparallel configuration. The microtubule binding unit of dynein/diffusive-NuMA can bind and diffuse along the microtubule. All motors have a finite unbinding rate from microtubule ends, i.e., dwell for some time before unbinding, a property required for microtubule end clustering. For simplicity, all motor types have the same rest length, spring constant, and diffusion constant. The simulations contained typically 5 to 20 motors per microtubule, leading to dense microtubule crosslinking and consistent sliding.

Detailed parameters of the model are listed in *SI Appendix*, Table S1, and Cytosim configuration files are provided at Zenodo (13993099).

## Quantification and Analysis

### Quantification of Number of Poles in Microtubule Networks.

#### Microtubule minus and plus end density maps.

First, we extracted the coordinates of microtubule minus and plus ends from the simulation data. We then created a two-dimensional (2D) grid with a resolution of 0.5 µm. For each type of microtubule end, a Gaussian peak with a width of 0.25 µm was generated, centered at the corresponding coordinates. These Gaussian peaks were summed across the final simulation time frames (frame number 950 to 1,000, corresponding to 31 to 33 min) to produce density maps. This approach resulted in a smoothed version of a 2D histogram. In the intensity maps, darker regions indicate higher density of microtubule ends. To enhance the visualization of areas with low intensity, we used a logarithmic scale for the color coding.

#### Microtubule end ratio density map.

We began by filtering out weak signals, i.e., any intensity values lower than 5% of the maximum intensity, from the microtubule minus end intensity map to exclude signals originating from single microtubules. We then calculated the ratio between the filtered minus end intensity and the plus end intensity using the formula *Ratio* = *(I_minus)/(1 + I_plus)*. The resulting ratio density plot was color-coded such that lighter colors represent higher ratios. This ratio map highlights regions where minus ends dominate, allowing pole detection. The color mapping for the ratio values is assigned as follows: black is used when the ratio is zero; for nonzero ratios, the “hot” Matplotlib color scheme is applied, scaling linearly with the ratio value. In the special case where I_plus = 0, the ratio is set to I_minus.

#### Pole detection and statistics.

Minus poles were detected from the local maxima in the microtubule end ratio density map, which were identified as points with higher density values than the surrounding grids. Poles were considered distinct if their density exceeded 8 and if they were at least 3 grids apart. This threshold was chosen to best separate the densities in the histogram into two distinct regimes. By counting the number of detected poles every 1.7 min, we generated a time series of the number of poles. The mean and the SD of the pole number were calculated for the last 13 min of the simulation, where the SD reflects the stability of the number of poles at steady state over time. The number of microtubule minus ends per pole was obtained by determining the number of minus ends in the pixel where a pole was detected. Since the simulations are stochastic, we averaged each quantity for 40 independent runs to obtain better statistics.

### Kymographs.

Kymographs were generated from regions of interest containing simulated image time series stacks using the Multi Kymograph FIJI plugin. The generated kymographs are two-dimensional images with vertical time axis and horizontal positional axis for the last 8 min of a 33 min long simulation. To produce microtubule “speckle” kymographs, this procedure was performed on images containing randomly placed points along the long axis of the microtubules. For other kymographs, the procedure was directly applied to microtubule plus ends, microtubule minus ends, and plus and minus motors, respectively.

### Crosslink Types.

To characterize the various types of connections made by motors between microtubules, we define five types of crosslinks: two types of H links, X links, V links, and T links, as previously described (20). H links connect parallel or antiparallel microtubules. X links connect crossing microtubules, V links connect two microtubule ends, where the “end” is defined as a length segment of 0.03 µm from the very microtubule end, and T links connect one microtubule end with the side of another microtubule.

### Spindle Width.

The spindle width was measured visually from the 2D projection of a simulated network at the midpoint between the poles, perpendicular to the long axis, at the end of the simulation and averaged across ten independent simulations.

## Supplementary Material

Appendix 01 (PDF)

Movie S1. **Kinesin-5 and a symmetrical minus motor can organize bipolar spindles via a monopolar intermediate.** Simulated microtubule network organized by N_+_ = 3200 kinesin-5 (red) and N_-_ = 800 - 6400 symmetrical minus motors (yellow), as indicated, forming a multipolar (top two), bipolar (bottom left), or monopolar network (bottom right). Both motors consist of two motor units (symbol + or –) with a speed v+ and v_ of 30 nm/s, but have opposite directionality. Microtubule growth speed is v_g_ = 30 nm/s. Conditions as in Fig. 2A–D. Time stamp is min:s.

Movie S2. **Kinesin-5 and dynein/diffusive-NuMA robustly generate bipolar spindles.** Simulated microtubule networks organized by N_+_ = 3200 kinesin-5 (red) and N_-_ = 3200 dynein/diffusive-NuMA motors (blue). Microtubule growth speed v_g_ = 30 nm/s. Condition as in Fig. 4B. Four different simulations are shown for the same condition. Time stamp is min:s.

Movie S3. **Microtubule end dynamics and microtubule flux in a bipolar spindle organized by kinesin-5 and dynein/diffusive-NuMA.** Simulated microtubule network organized by N_+_ = 3200 kinesin-5 (red) and N_-_ = 3200 dynein/diffusive-NuMA motors (blue). Microtubule growth speed v_g_ = 30 nm/s. Condition as in Fig. 4B and Movie 2. Features of the same spindle are shown separately, from top to bottom: plus (red) and minus (blue) motors; growing (green) and shrinking (red) plus ends; minus ends; microtubule speckles. Time stamp is min:s.

Movie S4. **Kinesin-5 and dynein/diffusive-NuMA organize bipolar spindles also when microtubules grow fast.** Simulated microtubule networks organized by N_+_ = 3200 kinesin-5 (red) and N_-_ = 3200 dynein/diffusive-NuMA motors (blue). Microtubule growth speed v_g_ = 120 nm/s. Conditions as in Fig. 5B. Four different simulations are shown. for the same condition. Time stamp is min:s.

Movie S5. **Kinesin-5 and dynein/diffusive-NuMA organize bipolar spindles within a range of minus motor numbers.** Simulated microtubule networks organized by N_+_ = 3200 kinesin-5 (red) and N_-_ = 800 - 3200 dynein/diffusive-NuMA motors (blue), as indicated. Microtubule growth speed v_g_ = 120 nm/s. Conditions as in Fig. 5D. Time stamp is min:s.

Movie S6. **Microtubule end dynamics and microtubule flux in a bipolar spindle organized by kinesin-5 and dynein/diffusive-NuMA at fast growth speed.** Simulated microtubule network organized by N_+_ = 3200 kinesin-5 and N_-_ = 3200 dynein/diffusive-NuMA motors. Microtubule growth speed v_g_ = 120 nm/s. Condition as in Fig. 5A and Movie 4. Features of the same spindle are shown separately, from top to bottom: plus (red) and minus (blue) motors; growing (green) and shrinking (red) plus ends; minus ends; microtubule speckles. Time stamp is min:s.

Movie S7. **Kinesin-5 and dynein/diffusive-NuMA organize a bipolar spindle in cylindrical box.** Simulated microtubule network organized by 19000 kinesin-5 (red) and dynein/diffusive-NuMA motors (blue) in a cylindrical container with 50 μm length and 5 μm diameter. Microtubules nucleate in the center of the cylinder within a small cylindrical volume of 1 μm length and 1 μm diameter at a rate of 16/s. Microtubule growth speed v_g_ = 120 nm/s. Condition is the same as in last row of Fig. 6C. Time stamp is min:s.

## Data Availability

Simulation file data have been deposited in zenodo (13993099) (54).
