# Supplementary material for: Molecular design principles for bipolar spindle organization by two opposing motors
Source: Proc Natl Acad Sci U S A. 2025 Mar 21;122(12):e2422190122. doi: 10.1073/pnas.2422190122 (PMC11962486; doi:10.1073/pnas.2422190122)
Supplement: Supplementary file 1 — Appendix 01 (PDF) [file pnas.2422190122.sapp.pdf]

Supplementary information for
Molecular design principles for bipolar spindle organization by two opposing motors

Wei-Xiang Chew[1], François Nédélec[2,#], Thomas Surrey[1,3,4,#]

[1] Centre for Genomic Regulation (CRG), Barcelona Institute of Science and Technology (BIST), Dr Aiguader 88, 08003 Barcelona, Spain
[2] Sainsbury Laboratory, University of Cambridge, 47 Bateman Street, Cambridge, CB2 1LR, UK
[3] Universitat Pompeu Fabra (UPF), Barcelona, Spain.
[4] Catalan Institution for Research and Advanced Studies (ICREA), Passeig de Lluis Companys 23, 08010 Barcelona, Spain

[#] Correspondence: thomas.surrey@crg.eu, fjn28@cam.ac.uk

This file includes:
Figures S1 to S8
Tables S1 & S2
Legends for Movies S1 to S6
Supplementary references

SUPPLEMENTARY FIGURES

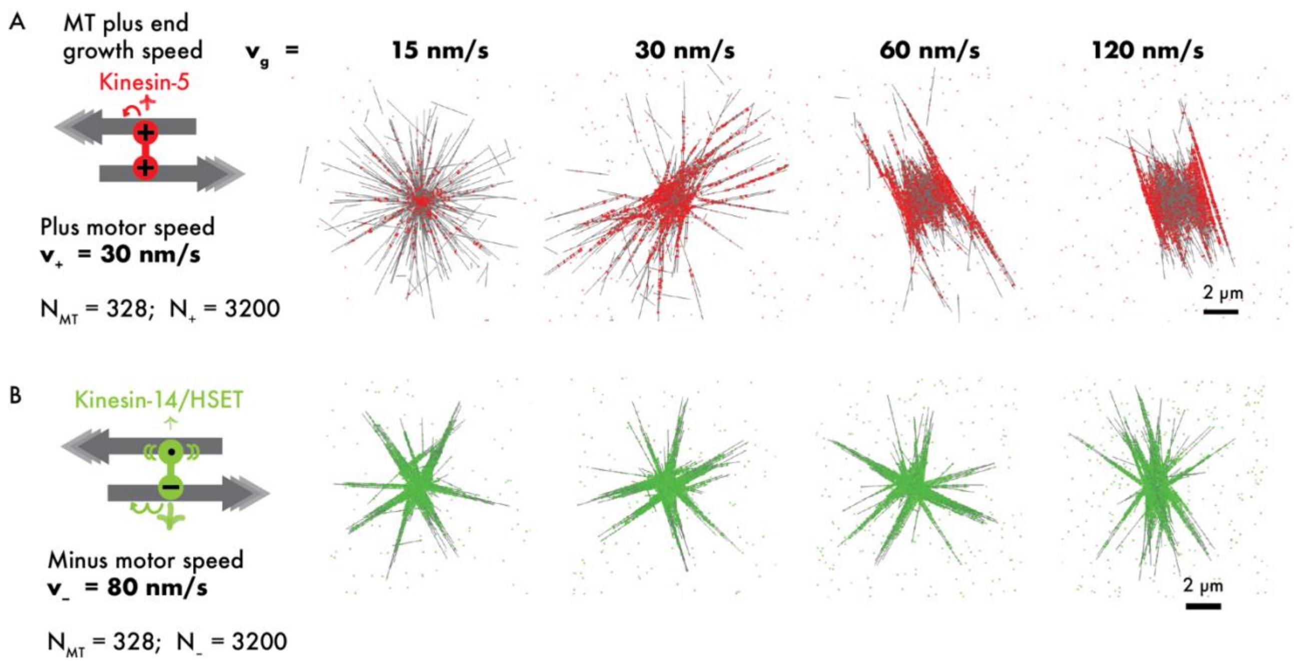

Figure S1. Networks generated by either kinesin-5 alone or HSET alone at different microtubule growth speeds. Microtubules (with average length L_{MT} = 2.5 μm, average number N_{MT} = 328) nucleate in a 1×1×0.2 μm^3 nucleation volume, as in Fig. 1, and grow with speeds v_g = 15, 30, 60 and 120 nm/s (left to right), as indicated, in the presence of (**A**) N_+ = 3200 kinesin-5, or **(B)** N_- = 3200 kinesin-14/HSET motors. Motor properties are the same as in Fig. 1. Microtubule mean length was kept constant by varying the catastrophe rate: 0.012, 0.024, 0.048, and 0.096/s (left to right), as the growth speed was varied. The steady state number of microtubules was kept constant by additionally varying the microtubule nucleation rate: 2, 4, 8 and 16/s (left to right) to compensate for the shorter microtubule lifetimes at higher microtubule growth speeds. Nematic networks in (A) were extensile for growth speeds v_g = 30—120 nm/s, as microtubules slid with kinesin-5 speed of 30 nm/s. For a summary of parameters, see Table S2.

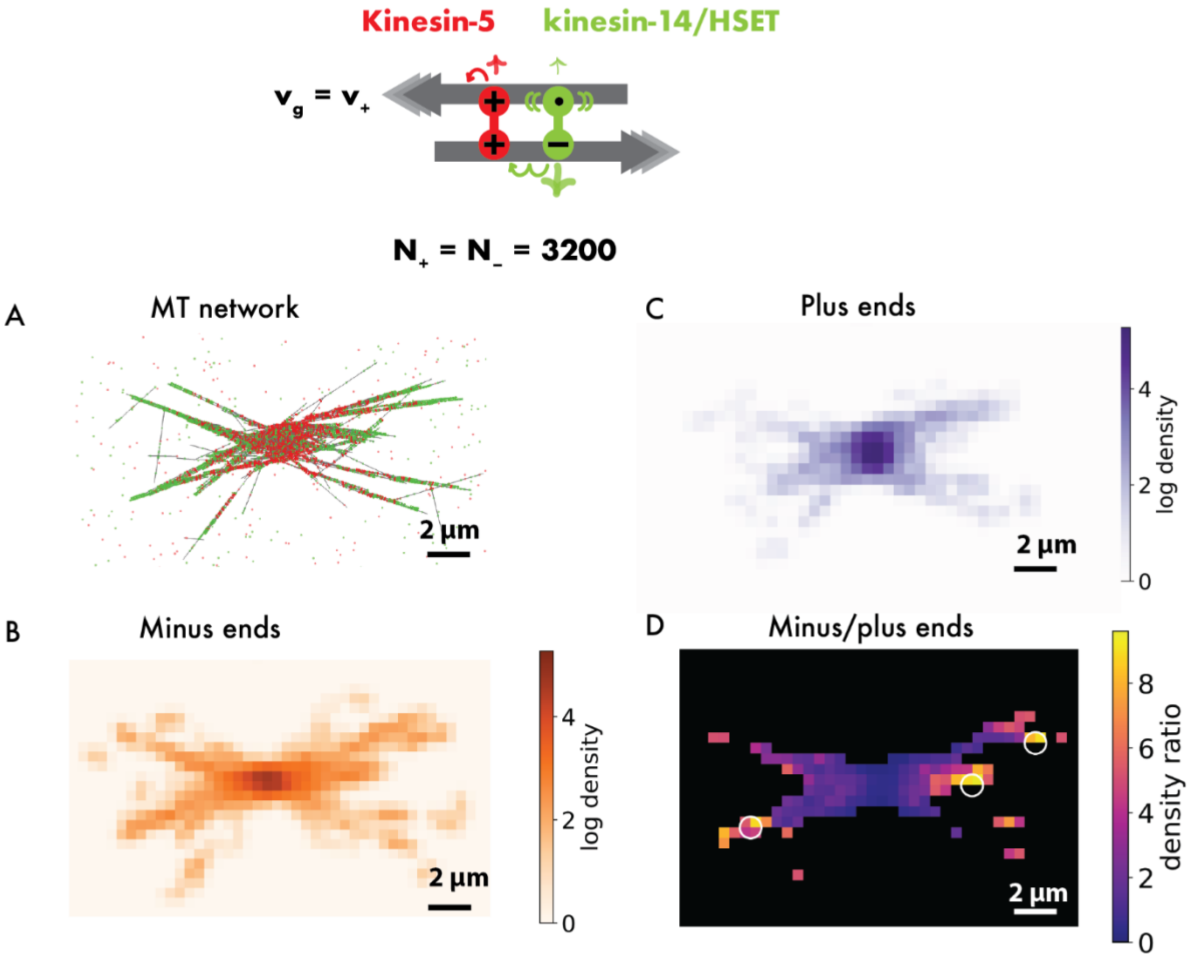

Figure S2. Microtubule end density map for a microtubule network organized by kinesin-5 and HSET. (A) Microtubule network formed by kinesin-5 (red) and HSET (green) at 32 minutes. Same network as shown in Fig. 1D. **(B)** Corresponding microtubule minus end density map (orange) and **(C)** microtubule plus end density map (blue), for the 31—33-minute interval, with density color-coded on a logarithmic scale. **(D)** Microtubule end ratio density map obtained from the microtubule end densities (B) & (C) (see Methods). Detected poles are indicated by circles (see Methods). For a summary of parameters, see Table S2.

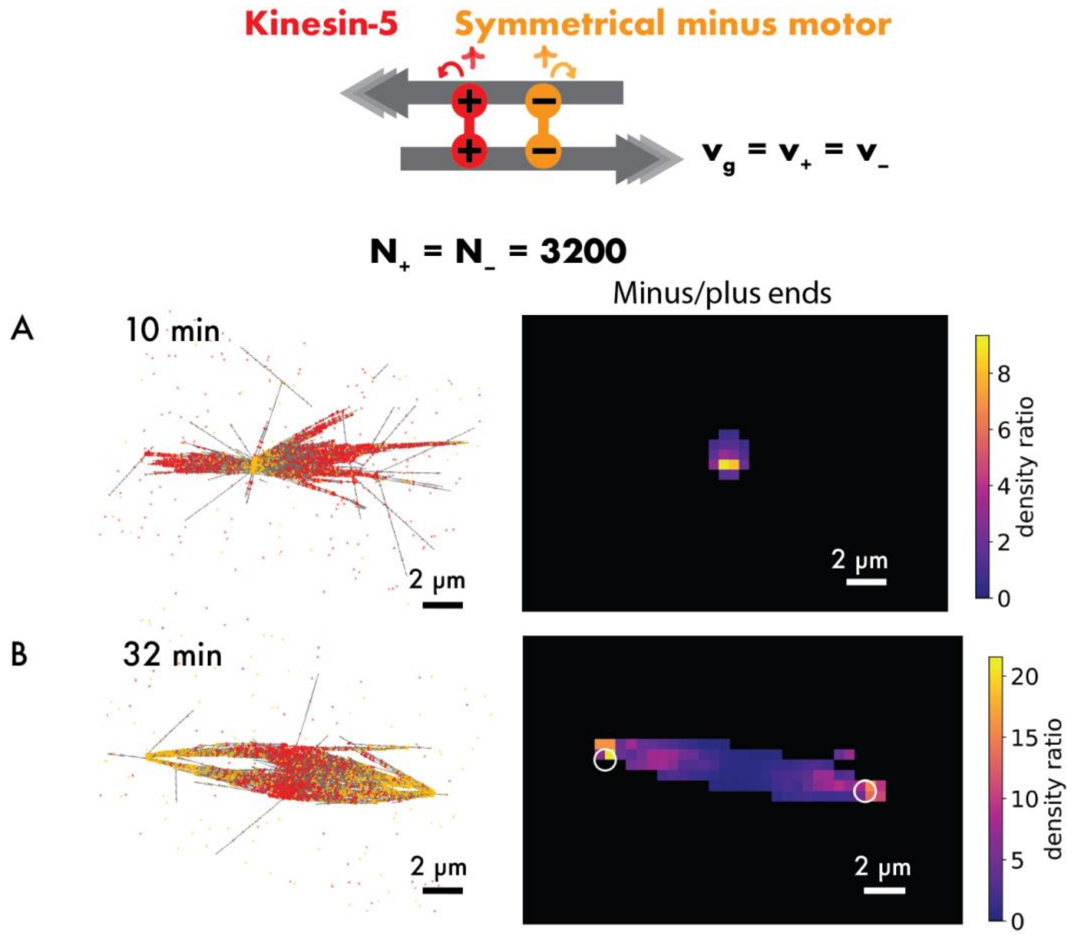

Figure S3. Microtubule end density maps of networks organized by kinesin-5 and a symmetrical minus motor when microtubules grow at kinesin-5 speed. *Top:* Schematic of the motors with properties as described for Fig. 2A. Microtubule plus-end growth speed and motor speeds are all 30 nm/s. Same microtubule network as in Fig. 2B at **(A)** 10 minutes and **(B)** 32 minutes after the start of self-organization, showing on the left the localization of kinesin-5 (red) and the symmetrical minus motor (yellow), and on the right the corresponding microtubule end ratio density maps. Circles indicate detected minus poles. For a summary of parameters, see Table S2.

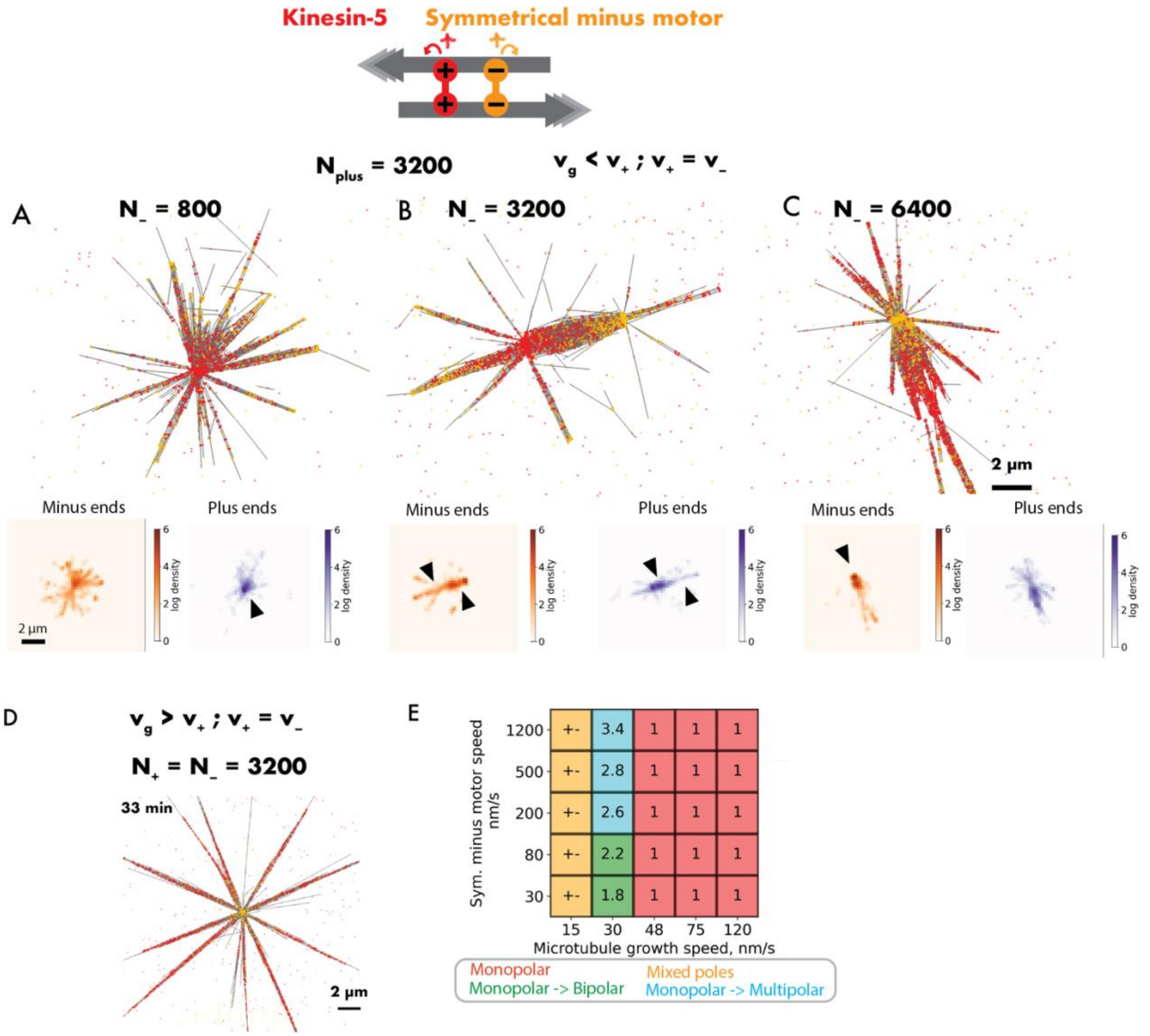

Figure S4. Networks generated by kinesin-5 and a symmetrical minus motor when the microtubule growth speed is slower or faster than the kinesin-5 speed. *Top:* Schematic of the motors with properties as described in Fig. 2A. **(A-C)** Images of microtubule networks formed after 32 minutes by $N_+ = 3200$ kinesin-5 (red) and $N_- = 800$ (A), 3200 (B), and 6400 (C) symmetrical minus motors (yellow) with microtubules growing at a speed of $v_g = 15$ nm/s, i.e., slower than both the plus and minus motor speeds of 30 nm/s. The corresponding density maps (bottom) show microtubule plus-end (blue) and minus-end (orange) densities. Detected poles are marked by triangles. Each simulation contains $N_{MT} = 358$ microtubules of $L_{MT} = 2.5$ µm in a box of size = $30 \times 30 \times 0.2$ µm^3. **(D)** Image showing the steady-state microtubule network organization by $N_+ = 3200$ kinesin-5 and $N_- = 3200$ symmetrical minus motors when the microtubule growth speed $v_g = 120$ nm/s is faster than motor speeds ($v_+ = v_- = 30$ nm/s) (one of the conditions shown in Fig. 2H in red). **(E)** Phase diagram of microtubule network organization for various combinations of symmetrical minus motor speed and microtubule growth speed. Numbers in the grid indicate the mean number of minus poles, with colors representing four states: monopolar to multipolar,

monopolar, monopolar to bipolar, and coexisting plus and minus poles (+ -). Each parameter set was simulated 30 times. Each simulation contains N_{MT} = 179 microtubules with an average length of L_{MT} = 5 µm in a box of size 50×50×0.2 µm^3. For a summary of parameters, see Table S2.

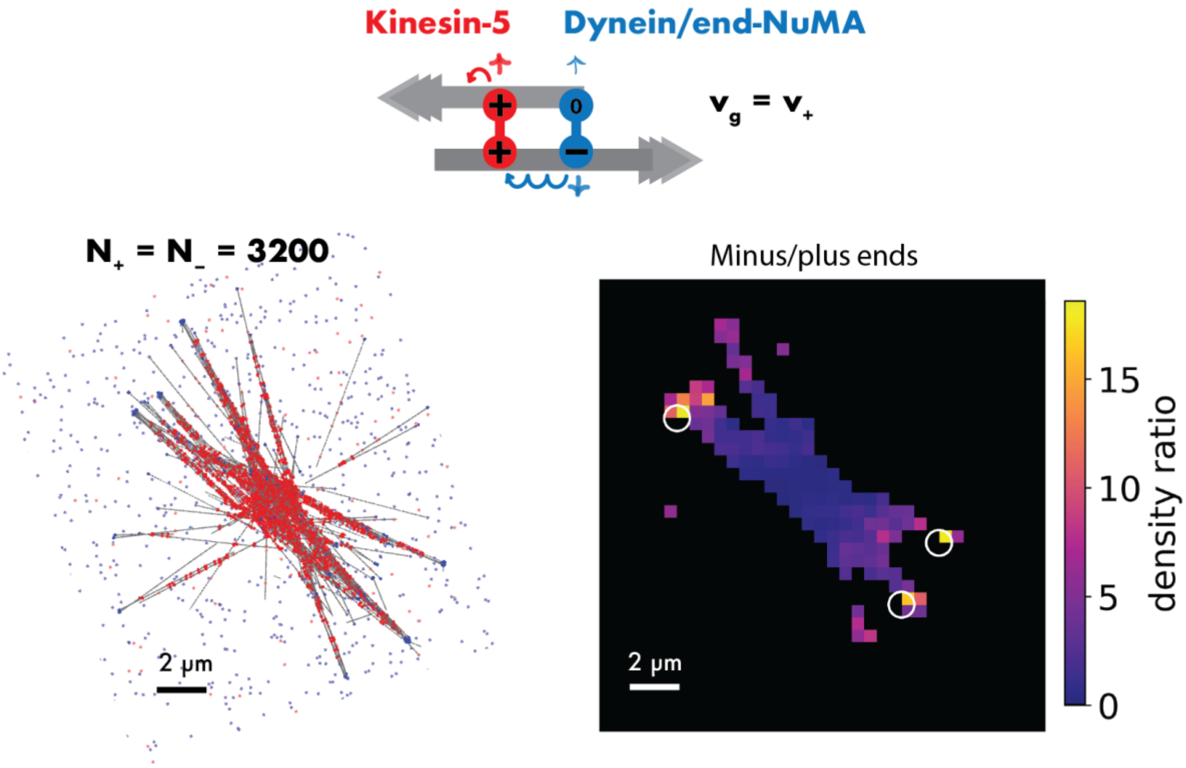

Figure S5. Microtubule end ratio density map for a network generated by kinesin-5 and dynein/end-NuMA. *Top:* Schematic of kinesin-5 (red) and dynein/end-NuMA motor (blue) with motor properties and microtubule dynamics properties (v_g = 30 nm/s) as in Fig. 3. *Bottom left:* Image of a simulated microtubule network organized by N_+ = 3200 kinesin-5 (red) and N_- = 3200 dynein/end-NuMA motors (blue) at 32 minutes. *Bottom right*: The corresponding microtubule end ratio density map with detected poles marked by circles. For a summary of parameters, see Table S2.

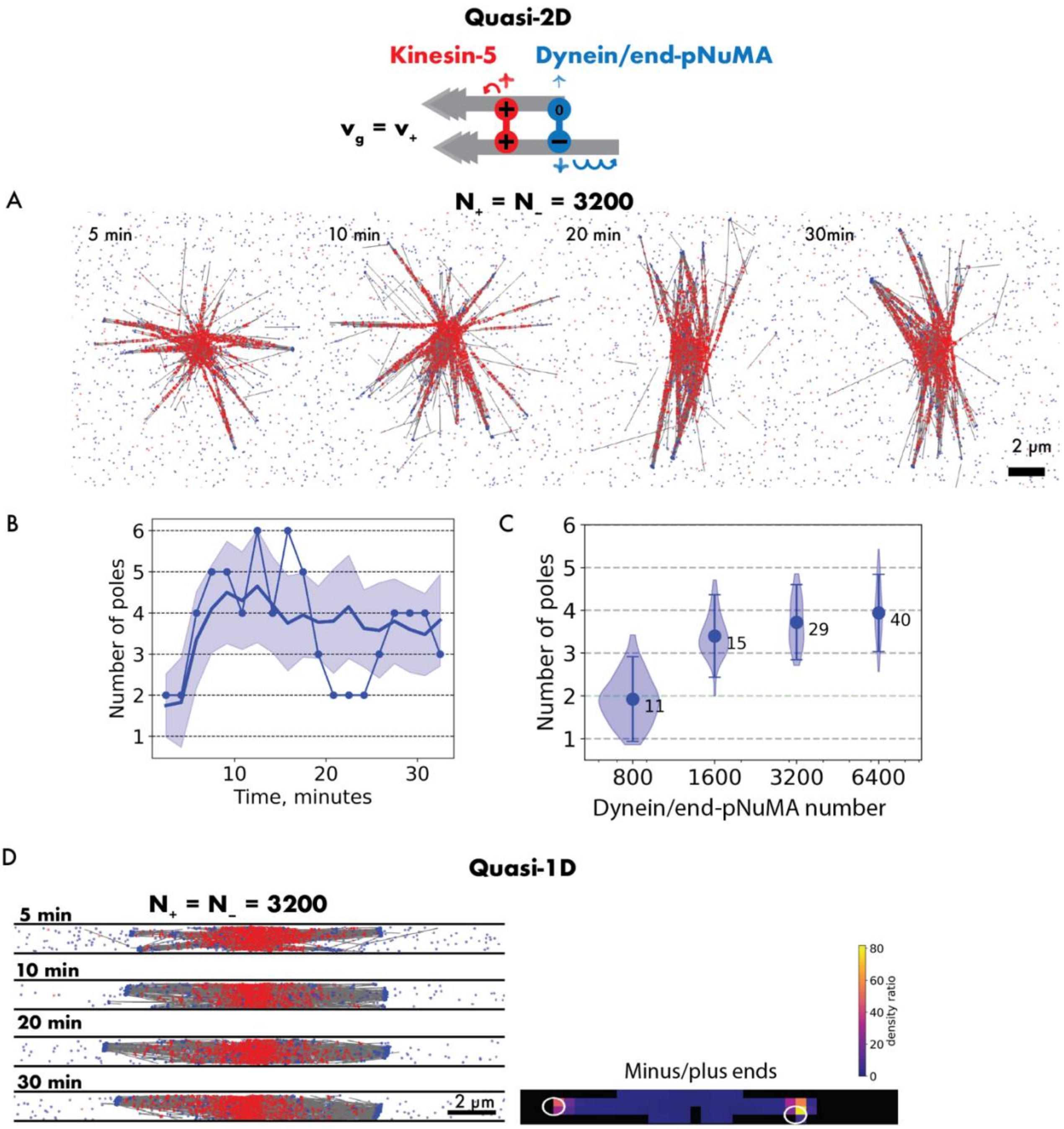

Figure S6. Kinesin-5 and dynein/end-NuMA with parallel microtubule binding selectivity can organize a bipolar spindle only in a narrow channel. *Top:* Schematic of kinesin-5 (red) and a dynein/end-NuMA variant (blue, dynein/end-pNuMA) that in contrast to dynein/end-NuMA in Fig. 3 connects only parallel microtubules. Microtubule dynamics as in Fig. 3 (v_g = 30 nm/s). **(A)** Images of a simulated microtubule network in a box of size 30×30×0.2 μm^3 organized around a 1x1x0.2 μm^3 nucleation volume by N_+ = 3200 kinesin-5 and N_- = 3200 dynein/end-pNuMA motors at the indicated times. **(B)** Time series showing the number of poles (connected dots) in the network shown in (A), the mean number of poles from N = 40 simulations (solid line) and the standard deviation (shaded region). **(C)** Steady-state pole statistics of various networks organized by N_+ = 3200 kinesin-

5 and N_- = 800 - 6400 dynein/end-pNuMA motors, as indicated: violin plot showing the distribution of the number of poles (shaded area), mean pole number (circle), mean standard deviation of pole number fluctuations over time (error bars), and the mean number of microtubules per pole (number next to circle). **(D)** *Left*: Images of a simulated microtubule network in a channel of dimensions 30×1×0.2 μm^3 organized around a 1x1x0.2 μm^3 nucleation volume by N_+ = 3200 kinesin-5 and N_- = 3200 dynein/end-pNuMA motors at the indicated times. Microtubule dynamics as in (A) *Right*: The corresponding microtubule end ratio density map at the end of the simulation with detected poles marked by circles. For a summary of parameters, see Table S2.

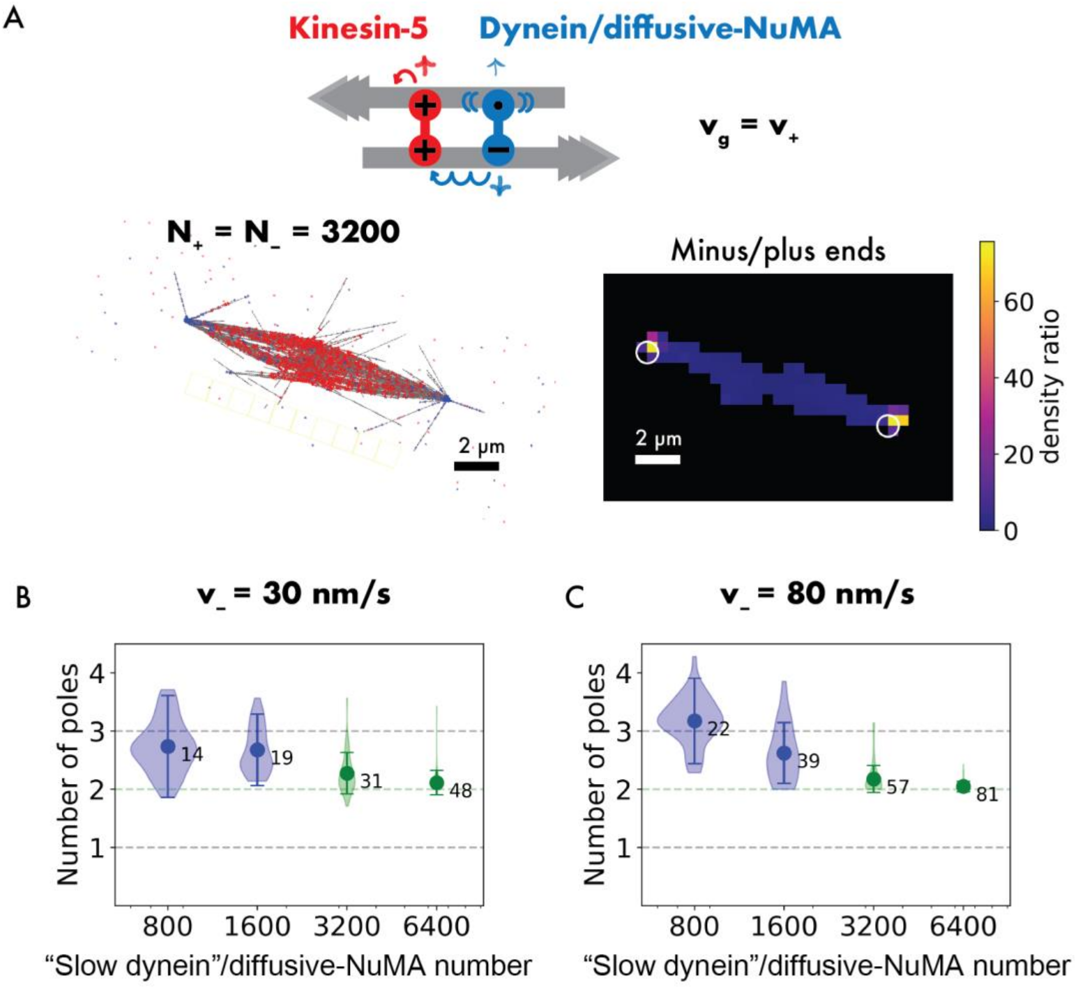

Figure S7. Kinesin-5 and dynein/diffusive-NuMA also organize bipolar spindles with slower dynein motor. (A) Images of a simulated microtubule network under conditions as in Fig. 4B after 32 minutes, with microtubules growing at v_g = 30 nm/s and organized by N_+ = 3200 kinesin-5 and N_- = 3200 dynein/diffusive-NuMA motors, showing on the left the motor distribution and on the right the microtubule end ratio density map with detected poles marked by circles. **(B, C)** Steady-state pole statistics of networks with N_+ = 3200 kinesin-5 and N_- = 800 - 6400 "slow dynein"/diffusive-NuMA motors having reduced minus motor speed of v_- = 30 nm/s (B) or v_- = 80 nm/s (C): violin plots showing the distribution of the number of poles (shaded area), mean pole number (circle), mean standard deviation of pole number fluctuations over time (error bars), and the mean number of microtubules per pole (number next to circle). Color code as in main figures. For a summary of parameters, see Table S2.

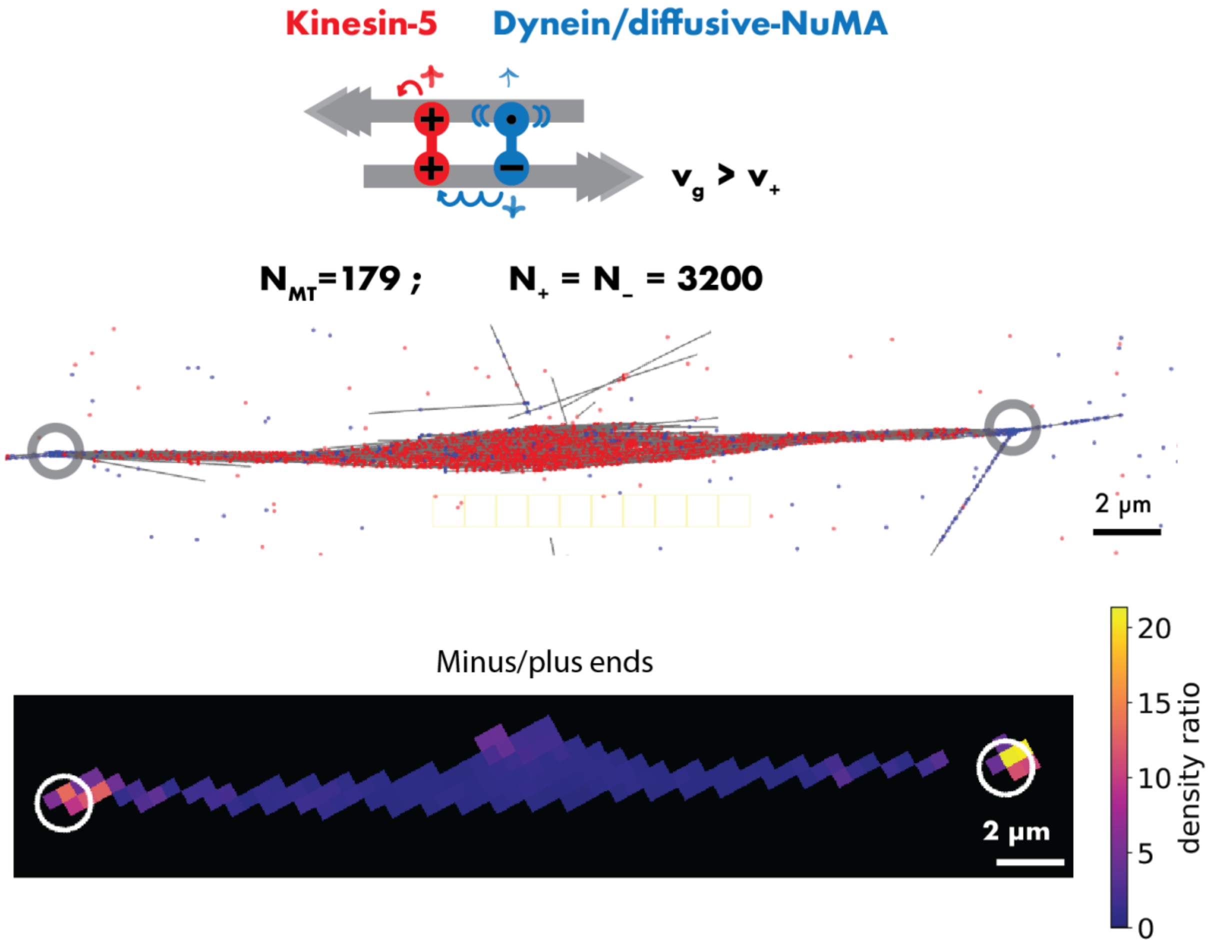

Figure S8. Kinesin-5 and dynein/diffusive-NuMA organize a bipolar spindle also with fast growing microtubules. Images of a simulated microtubule network under conditions as in Fig. 5B after 32 minutes, with microtubules growing at v_g = 120 nm/s and organized by N_+ = 3200 kinesin-5 and N_- = 3200 dynein/diffusive-NuMA motors, showing the motor distribution and microtubule end ratio density map with detected poles marked by circles. For a summary of parameters, see Table S2.

Table S1. Key parameters used in Cytosim computer simulations.

Parameter	Value	Note
**Simulation**		
time step	0.005 s	Small enough for convergence. Total time ~ 30 min
viscosity	1 pN s/μm^2	effective viscosity of the cytoplasm
box thickness	0.2 μm	thick enough for filament crossing
box width	30−50 μm	~12x L_{MT}
**Microtubule**		
growing speed v_g	0.03−0.12 μm/s	in vitro to physiological values
catastrophe rate	0.024/s	parameter controlling filament's length and lifetime
rescue rate	0.2/s	parameter controlling filament's length and lifetime
shrinking speed	0.5 μm/s	as measured (1)
mean length L_{MT}	2.5-5 μm	$v_g v_s/(v_s k_{cat}-v_g k_{res})$
life time τ_{MT}	~1 minute	$(v_g+v_s)/(v_s k_{cat}-v_g k_{res})$
steric distance	0.025 μm	~microtubule's diameter
steric force constant	50 pN/μm	Constrained by strength of thermal fluctuation and force of motors
rigidity	30 pN μm2	as measured (2)
lattice size	0.008 μm	tubulin dimer's length
nucleation rate	2−4/s	to maintain N_{MT} = 358 or total MT length of 895 μm
**Crosslinking motor**		
rest length	0.105 μm	0.025 μm (microtubule's diameter) + 0.08 μm (actual crosslinker's length)
link stiffness	100 pN/μm	Typical range
diffusion constant (when unbound)	10 μm^2/s	fast enough to have uniform distribution
**Kinesin-5**		
motor speed v_+	0.03 μm/s	as measured (3)
binding rate	0.5/s	estimated
unbinding rate k_{off+}	0.1/s	as measured (4)
binding length	0.12 μm	1.14x rest length
stall force	2 pN	within measured range (5)
**HSET motor**		
motor speed v_-	0.08 μm/s	within measured range (3, 6, 7)
binding rate	5/s	estimated
unbinding rate k_{off-}	5/s	as measured (8)
binding length	0.12 μm	1.14x rest length
stall force	2 pN	within measured range (5)

**HSET tail**		
diffusion constant (bound to microtubule)	0.1 $\mu m^2/s$	as measured (8)
binding rate	0.5/s	estimated
unbinding rate k_{offd}	0.01/s	as measured (8)
binding length	0.12 µm	1.14x rest length
**Symmetric motor**		
motor speed v_-	0.03−0.12 µm/s	parameter
binding rate	0.5/s	same as kinesin-5
unbinding rate k_{off-}	0.1/s	same as kinesin-5
binding length	0.12 µm	1.14x rest length
stall force	2 pN	same as kinesin-5
**Dynein motor**		
motor speed v_-	1.2 µm/s	as measured (9)
binding rate	0.1/s	estimated
unbinding rate k_{off-}	0.1/s	Same as kinesin motor
binding length	0.12 µm	1.14x rest length
stall force	2 pN	within measured range (5)
**NuMA**		
diffusion constant (bound to microtubule)	0.1 $\mu m^2/s$	as measured (10)
binding rate	0.5/s	estimated
unbinding rate k_{offn}	0.01/s	Same as HSET's tail
binding length	0.12 µm	1.14x rest length

Table S2. Microtubule and motor parameters in each figure.

**Figure**	1,S2	2A-G, S3,S4E	2H, S4D	3,S5	4	S7	5	S8	6	S1	S4A-C	S4E	S6A-C	S6D
**Simulation box** (μm^3)	30x30x0.2	30x30x0.2	50x50x0.2	30x30x0.2	30x30x0.2		50x50x0.2		50x50x0.2	30x30x0.2	30x30x0.2		30x30x0.2	30x1x0.2
**Microtubules**														
growth speed (nm/s)	30	30	120	30	30		120		120	15-120	15	15-120	30	30
shrinkage speed (nm/s)	500													
catastrophe rate (s-1)	0.024	0.024	0.061	0.024	0.024		0.061		0.061	0.012-0.096	0.012	0.012-0.096	0.024	0.024
rescue rate (s^{-1})	0.2													
mean length (μm)	2.5	2.5	5	2.5	2.5		5		5	2.5	2.5		2.5	2.5
life time (min)	1.5	1.5	1.6	1.5	1.5		1.6		1.6	1.5	1.5		1.5	1.5
number	358	358	179	358	358		179		179-1432	358	358		358	358
**Kinesin-5**														
number	3200													
speed (nm/s)	30													
unbinding rate (s^{-1})	0.1													
**Minus motor**														
number	1600-12800	800-6400		800-6400	800-6400		800-6400		3200- 19000	3200	800-6400		800-6400	3200
first unit	HSET motor	sym. motor		dynein motor	dynein motor		dynein motor		dynein motor	HSET motor	sym. motor		dynein motor	dynein motor
speed (nm/s)	80	30-1200	30	1200	30-1200	1200	30-1200	1200	1200	80	30	30-1200	1200	1200
unbinding rate (s^{-1})	5	0.1		0.1	0.1-5	0.1-5	0.1	0.1	0.1	5	0.1		0.1	0.1
second unit	HSET tail	sym. motor		end NuMA	diffusive NuMA		diffusive NuMA		diffusive NuMA	HSET tail	sym. motor		end pNuMA	end NuMA
speed (nm/s) or diffusion constant ($\mu m^2/s$)	0.1 $\mu m^2/s$	30-1200 nm/s	30 nm/s	0	0.1 $\mu m^2/s$		0.1 $\mu m^2/s$		0.1 $\mu m^2/s$	0.1 $\mu m^2/s$	30 nm/s		0	0
unbinding rate (s^{-1})	0.01	0.1		0.01	0.01		0.01		0.01	0.01	0.1		0.01	0.01

LEGENDS FOR MOVIES

Movie S1. Kinesin-5 and a symmetrical minus motor can organize bipolar spindles via a monopolar intermediate. Simulated microtubule network organized by N_+ = 3200 kinesin-5 (red) and N_- = 800 - 6400 symmetrical minus motors (yellow), as indicated, forming a multipolar (top two), bipolar (bottom left), or monopolar network (bottom right). Both motors consist of two motor units (symbol + or –) with a speed v_+ and v_- of 30 nm/s, but have opposite directionality. Microtubule growth speed is v_g = 30 nm/s. Conditions as in Fig. 2A-D. Time stamp is min:s.

Movie S2. Kinesin-5 and dynein/diffusive-NuMA robustly generate bipolar spindles. Simulated microtubule networks organized by N_+ = 3200 kinesin-5 (red) and N_- = 3200 dynein/diffusive-NuMA motors (blue). Microtubule growth speed v_g = 30 nm/s. Condition as in Fig. 4B. Four different simulations are shown for the same condition. Time stamp is min:s.

Movie S3. Microtubule end dynamics and microtubule flux in a bipolar spindle organized by kinesin-5 and dynein/diffusive-NuMA. Simulated microtubule network organized by N_+ = 3200 kinesin-5 (red) and N_- = 3200 dynein/diffusive-NuMA motors (blue). Microtubule growth speed v_g = 30 nm/s. Condition as in Fig. 4B and Movie 2. Features of the same spindle are shown separately, from top to bottom: plus (red) and minus (blue) motors; growing (green) and shrinking (red) plus ends; minus ends; microtubule speckles. Time stamp is min:s.

Movie S4. Kinesin-5 and dynein/diffusive-NuMA organize bipolar spindles also when microtubules grow fast. Simulated microtubule networks organized by N_+ = 3200 kinesin-5 (red) and N_- = 3200 dynein/diffusive-NuMA motors (blue). Microtubule growth speed v_g = 120 nm/s. Conditions as in Fig. 5B. Four different simulations are shown. for the same condition. Time stamp is min:s.

Movie S5. Kinesin-5 and dynein/diffusive-NuMA organize bipolar spindles within a range of minus motor numbers. Simulated microtubule networks organized by N_+ = 3200 kinesin-5 (red) and N_- = 800 - 3200 dynein/diffusive-NuMA motors (blue), as indicated. Microtubule growth speed v_g = 120 nm/s. Conditions as in Fig. 5D. Time stamp is min:s.

Movie S6. Microtubule end dynamics and microtubule flux in a bipolar spindle organized by kinesin-5 and dynein/diffusive-NuMA at fast growth speed. Simulated microtubule network organized by N_+ = 3200 kinesin-5 and N_- = 3200 dynein/diffusive-NuMA motors. Microtubule growth speed v_g = 120 nm/s. Condition as in Fig. 5A and Movie 4. Features of the same spindle are shown separately, from top to bottom: plus (red) and minus (blue) motors; growing (green) and shrinking (red) plus ends; minus ends; microtubule speckles. Time stamp is min:s.

Movie S7. Kinesin-5 and dynein/diffusive-NuMA organize a bipolar spindle in cylindrical box. Simulated microtubule network organized by 19000 kinesin-5 (red) and dynein/diffusive-NuMA motors (blue) in a cylindrical container with 50 µm length and 5 µm diameter. Microtubules nucleate in the center of the cylinder within a small cylindrical volume of 1 µm length and 1 µm diameter at a rate of 16/s. Microtubule growth speed v_g = 120 nm/s. Condition is the same as in last row of Fig. 6C. Time stamp is min:s.

SUPPLEMENTARY REFERENCES

1. R. A. Walker *et al.*, Dynamic instability of individual microtubules analyzed by video light microscopy: rate constants and transition frequencies. *J Cell Biol* **107**, 1437-1448 (1988).
2. M. Dogterom, B. Yurke, Measurement of the force-velocity relation for growing microtubules. *Science* **278**, 856-860 (1997).
3. J. Roostalu, J. Rickman, C. Thomas, F. Nédélec, T. Surrey, Determinants of Polar versus Nematic Organization in Networks of Dynamic Microtubules and Mitotic Motors. *Cell* **175**, 796-808.e714 (2018).
4. L. C. Kapitein *et al.*, The bipolar mitotic kinesin Eg5 moves on both microtubules that it crosslinks. *Nature* **435**, 114-118 (2005).
5. B. H. Blehm, T. A. Schroer, K. M. Trybus, Y. R. Chemla, P. R. Selvin, In vivo optical trapping indicates kinesin's stall force is reduced by dynein during intracellular transport. *Proc Natl Acad Sci U S A* **110**, 3381-3386 (2013).
6. K. Furuta, Y. Y. Toyoshima, Minus-end-directed motor Ncd exhibits processive movement that is enhanced by microtubule bundling in vitro. *Curr Biol* **18**, 152-157 (2008).
7. S. R. Norris *et al.*, Microtubule minus-end aster organization is driven by processive HSET-tubulin clusters. *Nat Commun* **9**, 2659 (2018).
8. M. Braun *et al.*, Changes in microtubule overlap length regulate kinesin-14-driven microtubule sliding. *Nat Chem Biol* **13**, 1245-1252 (2017).
9. L. Urnavicius *et al.*, Cryo-EM shows how dynactin recruits two dyneins for faster movement. *Nature* **554**, 202-206 (2018).
10. S. Forth, K. C. Hsia, Y. Shimamoto, T. M. Kapoor, Asymmetric friction of nonmotor MAPs can lead to their directional motion in active microtubule networks. *Cell* **157**, 420-432 (2014).
